# Co-ordinated shifts in deep-water formation and Gulf Stream migration during abrupt climate changes

**DOI:** 10.1038/s41467-026-73832-4

**Published:** 2026-06-11

**Authors:** Fangjingcheng Zhu, Alice Carter-Champion, Jack H. Wharton, Joel Bracamontes-Ramírez, Andrea Burke, Peter B. de Menocal, David Fairman, Lloyd D. Keigwin, Thomas M. Marchitto, Eirini Papachristopoulou, James W. B. Rae, Yair Rosenthal, Ning Zhao, David J. R. Thornalley

**Affiliations:** 1 Department of Geography, University College London, London, UK; 2 Centre for Quaternary Research, Department of Geography, Royal Holloway, University of London, London, UK; 3 Department of Earth System Sciences, University of Hamburg, Hamburg, Germany; 4 School of Earth and Environmental Sciences, University of St Andrews, St Andrews, UK; 5 Woods Hole Oceanographic Institution, Woods Hole, MA USA; 6 Department of Geological Sciences and INSTAAR, University of Colorado, Boulder, CO USA; 7 Department of Marine and Coastal Sciences, Rutgers University, New Brunswick, NJ USA; 8 Department of Earth and Planetary Sciences, Rutgers University, New Brunswick, NJ USA; 9 State Key Laboratory of Estuarine and Coastal Research and School of Marine Sciences, East China Normal University, Shanghai, China; 10 Present Address: School of Ocean and Earth Science, University of Southampton, Waterfront Campus, National Oceanography Centre, Southampton, UK

**Keywords:** Palaeoceanography, Physical oceanography, Palaeoclimate

## Abstract

Theory and models suggest the Gulf Stream may shift northwards under projected Atlantic Meridional Overturning Circulation weakening. Yet Gulf Stream behaviour during past abrupt cold events remains poorly constrained. Here we present high-resolution paleoceanographic records from the Northwest Atlantic during the last deglaciation. During the Younger Dryas cold period, we document a northward Gulf Stream shift evidenced from coherent surface and subsurface warming. Our sortable silt data suggest a strengthening of upper North Atlantic Deep Water that opposes weakening lower North Atlantic Deep Water, consistent with a seesaw feedback between the Nordic overflows and subpolar gyre. Our results constrain a co-ordinated sequence at the Younger Dryas onset: initial lower North Atlantic Deep Water weakening and subpolar sea‑ice expansion, lagged (58 ± 38 yr) by an increase in upper North Atlantic Deep Water and an eventual atmospheric reorganization (84 ± 51 yr after onset). These findings provide empirical support for model projections of future Gulf Stream shifts.

## Abstract (web summary)

Paleoceanographic reconstructions from the Northwest Atlantic reveal coordinated shifts in Gulf Stream position and upper and lower North Atlantic Deep Water formation during past abrupt climate events, offering insights for future climate change.

## Introduction

**Fig. 1:**
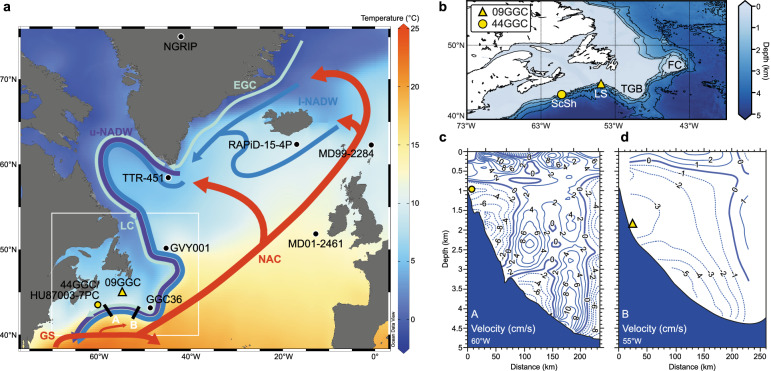
Locations of study sites and modern ocean conditions in the North Atlantic. **a** Map shows annual mean sea surface temperature^118^ (colour shading) and surface and deep circulations^20^ (arrows) in the North Atlantic. Locations of 44GGC (966 m) and HU87003-7PC (920 m) are shown in yellow circles. Location of 09GGC (1854 m) is shown in a yellow triangle. Other study sites discussed in this paper are shown as black circles: GGC36 (1520 m), GVY001 (3721 m), MD01-2461 (1153 m), TTR-451 (1927 m), RAPiD-15-4P (2133 m), MD99-2284 (1500 m), and North Greenland Ice Core Project (NGRIP). Black lines denote locations of the cross sections at 60 °W (A) and 55 °W (B) shown in panels (**c**, **d**), respectively. GS Gulf Stream, NAC North Atlantic Current, EGC East Greenland Current, LC Labrador Current, u/l-NADW upper/lower-North Atlantic Deep Water. The map was generated using Ocean Data View^119^. **b** Bathymetry map of the Northwest Atlantic, where locations of 44GGC and 09GGC are shown in yellow circle and triangle, respectively. FC Flemish Cap, TGB Tail of Grand Banks, LS Laurentian Slope, ScSh Scotian Shelf. Ocean bathymetry is from the GEBCO_2014 global bathymetric grid (30 arc-second resolution)^120^. **c** Absolute geostrophic velocity at 60°W^121^ (section A in **a**). Location of 44GGC is marked in a yellow circle. **d** Absolute geostrophic velocity at 55 °W^122,123^ (section B in **a**). The location of 09GGC is marked in a yellow triangle. In (**c**, **d**), positive values indicate poleward flow. Note both 44GGC and 09GGC are located in the core of the u-NADW flow.

The Atlantic Meridional Overturning Circulation (AMOC), comprising northward transport of warm surface water and southward return flow of cold North Atlantic Deep Water (NADW), plays a key role in redistributing heat, salt and nutrients in the climate system^1^. Today, NADW is composed of two distinct sources^2^: upper NADW (u-NADW) formed in the subpolar North Atlantic and lower NADW (l-NADW) formed by the two main overflows of Nordic Seas-derived waters across the Greenland-Scotland Ridge. Another key component of the North Atlantic circulation is the horizontal gyre circulation, composed of an anticyclonic subtropical gyre and a quasi-cyclonic subpolar gyre (SPG), both of which are important for meridional ocean heat transport^3^ and the densification of North Atlantic waters upon their transformation to NADW^4^. The vertical overturning and horizontal gyre circulation systems meet in the Northwest Atlantic, known as the pacemaker region for AMOC variability^1^. This region not only marks the boundary between the subtropical gyre and SPG, where the warm Gulf Stream meets the cold Labrador Current, but also where the southward-flowing Deep Western Boundary Current, a major conduit for exporting NADW, crosses underneath the Gulf Stream (Fig. 1a).

Under anthropogenic climate change, the Northwest Atlantic is warming at a rate significantly faster than the global average^5^, whilst a parallel cooling trend occurs in the subpolar North Atlantic^6^, forming a dipole pattern in sea surface temperature (SST)^7^. Modelling studies have attributed the dipole pattern to a northward shift in the Gulf Stream (causing the warming in the Northwest Atlantic) and a reduced northward ocean heat transport (linked to the cooling in the central subpolar North Atlantic), both linked to a slowdown of the AMOC^8,9^, whose current state has been suggested to be the weakest in the past millennium^10,11^. Furthermore, the AMOC may be subject to substantial weakening in the future^12,13^, potentially leading to abrupt changes partially analogous to rapid climate oscillations observed during the last glacial period^14,15^. However, the behaviour of the Gulf Stream location during these past abrupt climate events remains poorly studied due to the absence of high-quality records in this region. In addition, although previous studies have suggested a reorganisation in NADW during past abrupt climate change^16–18^, the relationship between changes in u-NADW and l-NADW and the position of the Gulf Stream in the Northwest Atlantic is not well constrained.

**Fig. 2:**
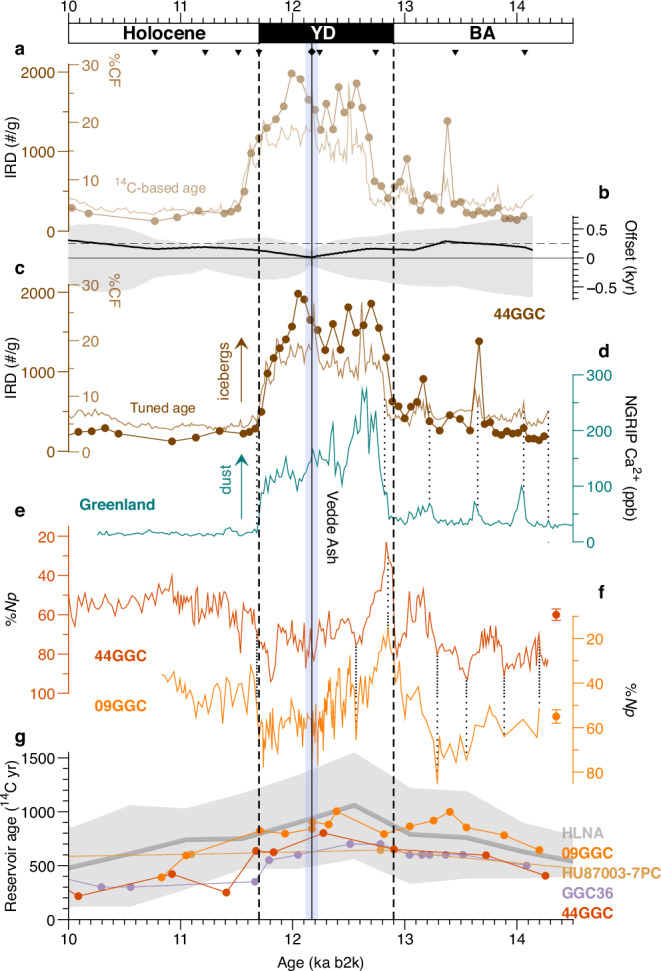
Radiocarbon-based and Tuned age models – linked to Greenland ice core chronology. **a** 44GGC %CF (light brown line) and IRD (dark brown line with dots) plotted on the Radiocarbon-based age model based on a tephra tie point (diamond) and ^14^C dates (inverted triangles) calibrated using the Marine20 calibration curve^82^, corrected with regional reservoir age (ΔR) estimated from Vedde Ash tephra layer with uncertainties to account for variable ΔR throughout the deglaciation. **b** Temporal offset between the Radiocarbon-based and Tuned age models of 44GGC (black line). Also shown is the uncertainty range of the Radiocarbon-based age model (grey shading; 95% confidence interval, CI). **c** Same as in (**a**) but plotted on the Tuned age model using the tephra tie point (vertical solid line) and the correlation of %CF and IRD with NGRIP Ca^2+^ (tie points indicated by dotted lines). **d** NGRIP Ca^2+^^21^. **e** 44GGC %*Np* plotted on the Tuned age model. **f** 09GGC %*Np* plotted on the Tuned age model synchronised with 44GGC using the tephra tie point (vertical solid line) and correlation with 44GGC %*Np* (tie points indicated by dotted lines). The error bars in (**e**, **f**) denote averaged ±1σ errors. **g** Surface ^14^C reservoir ages of 44GGC (red), 09GGC (orange) and HU87003-7PC (gold) calculated using the stratigraphic age constraints. Note the chronology of HU87003-7PC was synchronised with 44GGC by correlating *Np δ*^18^O (Supplementary Fig. 3a). Also shown is the regionally averaged high-latitude North Atlantic (HLNA) surface ^14^C reservoir age^27^ (thick grey line) with 95% CI (light grey shading). The regional surface reservoir ages were then applied to GGC36 (purple) and used for correcting its ^14^C dates^39^ (Supplementary Fig. 3d). The age of the Vedde Ash tephra layer is indicated in a solid vertical line with shading denoting ± 1σ error^21^.

Here we use three marine sediment cores with sub-centennial resolution (Fig. 1; KNR197-10-44GGC, 43°21.01’ N, 60°12.48’ W, 966 m, hereafter 44GGC; KNR158-4-09GGC, 44°49.60’ N, 54°53.78’ W, 1854 m, hereafter 09GGC; HU87003-7PC, 43°20.70’ N, 60°12.90’ W, 920 m) to study the paleoceanographic changes in the Northwest Atlantic prior to, and during, the Younger Dryas (YD), which marks an abrupt return from the warm Bølling–Allerød (BA) to near-glacial conditions in the North Atlantic region during the last deglaciation^19^. Today, these sites are located on the shoreward, northern side of the Gulf Stream (Fig. 1a) and are bathed at depth by u-NADW^20^ (Fig. 1c, d and Supplementary Fig. 1). Our records were synchronised with the Greenland ice core chronology^21^ (Methods; Fig. 2) by correlating the ice-rafted detritus (IRD) with the North Greenland Ice Core Project (NGRIP) Ca^2+^ (a proxy for dust)^21^, based on the assumption that the centennial- and millennial-scale IRD deposition events are associated with near-synchronous iceberg discharges from circum-North Atlantic ice sheets, controlled by rapid climatic/atmospheric oscillations^22–26^. The robustness of our age model is supported by the synchronisation of the Vedde Ash layer (Methods; Supplementary Fig. 2), and the agreement of our surface ^14^C reservoir ages (derived from combining planktic foraminiferal ^14^C dates with our Tuned age model) and the regional high-latitude North Atlantic reservoir age stack^27^ (Fig. 2).

In this article, we first reconstruct the deglacial changes in the strength of u-NADW using sortable silt grain size (SS), an established proxy for flow speed^28–30^. We then study the surface and subsurface temperature changes in the Northwest Atlantic and explore potential changes in the Gulf Stream^31^. Finally, we discuss broader implications for the North Atlantic circulation during abrupt climate change.

## Results and discussion

### Seesaw relationship between the upper- and lower-North Atlantic Deep Water

**Fig. 3:**
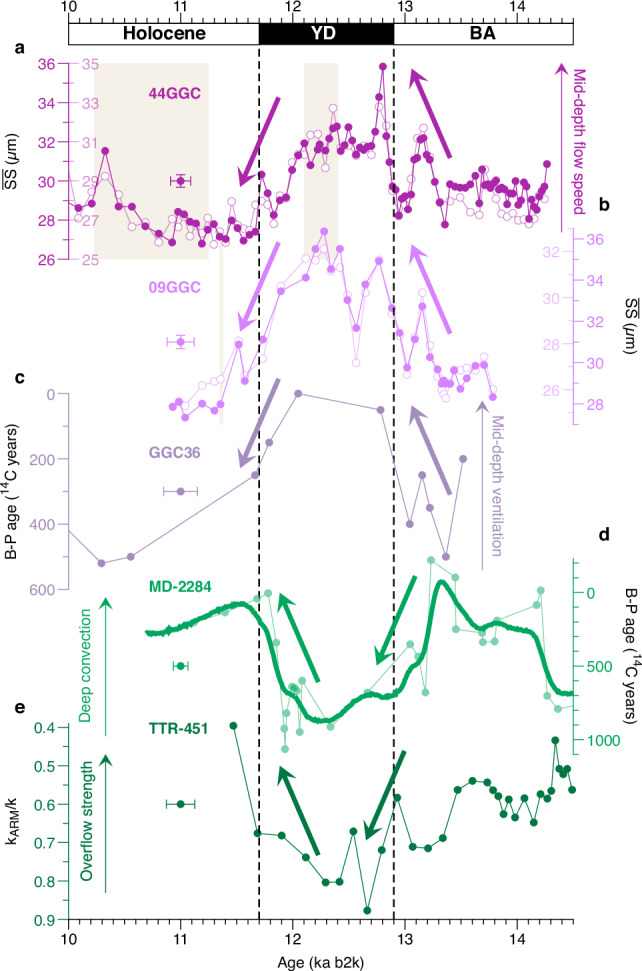
Seesaw relationship between the u-NADW and l-NADW during the last deglaciation. **a** 44GGC $\overline{{{\rm{SS}}}}$ reflecting mid-depth flow speed. **b** 09GGC $\overline{{{\rm{SS}}}}$ reflecting mid-depth flow speed. $\overline{{{\rm{SS}}}}$ data were measured on Coulter (line with solid dots) and Malvern (line with open dots). Light beige shadings denote intervals where $\overline{{{\rm{SS}}}}$ data do not pass the validity test^91^ (Supplementary Fig. 5), indicating poor sorting and unreliable records. **c** GGC36 B-P ^14^C age^39^ reflecting mid-depth ventilation. **d** MD99-2284 B-P ^14^C age^41^ reflecting Nordic Seas deep convection. **e** TTR-451 κ_ARM_/κ^42^ reflecting the strength of the Nordic Seas overflow (l-NADW). All published records are reported in their original age models except (**c**) GGC36, where the surface ^14^C reservoir age from the present study was applied to the ^14^C dates to account for regional reservoir effects (Fig. 2g; Supplementary Fig. 3d). In (**a**–**e**), horizontal floating error bars denote average age uncertainties ( ± 1σ). In (**a**,** b**), vertical floating error bars denote average analytical error ( ± 1σ).

The mean SS ($\overline{{{\rm{SS}}}}$) provides a quantitative estimate for the flow speed of near-bottom currents^29^. We use $\overline{{{\rm{SS}}}}$ of 44GGC and 09GGC to infer deglacial changes in the strength of u-NADW. There is good agreement between the $\overline{{{\rm{SS}}}}$ records of 44GGC and 09GGC during the deglaciation (Fig. 3a, b). Both records have low $\overline{{{\rm{SS}}}}$ during the early BA, followed by a sharp increase from ~13.2 to 12.8 ka. Applying the flow-speed calibration for SS^29^ suggests an increase from 15 ± 1 cm/s to 19 ± 2 cm/s in 44GGC and 15 ± 1 cm/s to 21 ± 2 cm/s in 09GGC during the transition from the early BA to early YD, suggesting an increase in u-NADW strength of 32 ± 12%. The $\overline{{{\rm{SS}}}}$ remains high during much of the YD, before declining after ~12.3 ka. Superimposed on the long-term increasing trend of $\overline{{{\rm{SS}}}}$ from BA to YD are a series of centennial-scale abrupt increases, centred at ~13.7, 13.2, and 12.8 ka.

An important consideration is whether our SS records reflect changes in basin-scale u-NADW strength rather than other oceanographic processes, such as slope-water variability, mesoscale recirculation, or eddy activity. Modern hydrographic data from the Northwest Atlantic (Supplementary Fig. 1) show that both 09GGC and 44GGC lie within the density layer occupied by u-NADW, rather than being influenced by deeply penetrating slope waters. Consistent with this, modern velocity sections (Fig. 1c, d) indicate that our sites lie within the core of the southward-flowing u-NADW within the Deep Western Boundary Current. An alternative explanation for the observed increase in $\overline{{{\rm{SS}}}}$ during the YD could be a strengthening of the northern recirculation gyre. However, model results suggest that a stronger recirculation gyre would shift the Gulf Stream southward^32^, opposite to the behaviour reconstructed from our records (see section below).

Another potential driver for the observed increase in our $\overline{{{\rm{SS}}}}$ records is an enhanced eddy kinetic energy^30^ given the proximity of strong frontal systems and associated baroclinic instability (Fig. 1a). As the Labrador Current and u-NADW act as a barrier for ocean eddies near the slope, an enhanced eddy activity would require a near-collapse of the Labrador Current and u-NADW^33^. However, this mechanism is inconsistent with a wide range of additional regional evidence that supports the prevailing paradigm^14^ of more vigorous u-NADW formation and ventilation during the YD: (1) Published $\overline{{{\rm{SS}}}}$ data also infer faster flow of u-NADW at ~1 km water depth at Flemish Pass^34^ and at 1.5–2 km water depth on the slope to the west and north of Flemish Cap^35,36^, as well as more vigorous flow of u-NADW (sometimes also termed glacial North Atlantic Intermediate Water) in the subtropical Northwest Atlantic^37^ and subpolar Northeast Atlantic^38^; (2) benthic-planktic foraminiferal radiocarbon data indicate better ventilated intermediate water at 1.5 km water depth at the Tail of the Grand Banks (Fig. 3c)^39^, and well-ventilated water above 2.3 km in the subtropical Northwest Atlantic^40^; (3) Cd/Ca data from the Bahama Banks indicate the enhanced production of nutrient-depleted North Atlantic intermediate water during the YD^18^. Taken together, these observations support the interpretation that our SS records are consistent with basin-scale variability in u-NADW strength rather than local circulation changes.

The inferred strengthening of u-NADW during the YD parallels a pronounced weakening of l-NADW (Fig. 3). For example, benthic-planktic radiocarbon ventilation ages show a significant weakening of Nordic Seas deep convection during the YD (Fig. 3d)^41^, while further downstream at Eirik Drift, magnetic grain size (κ_ARM_/κ) data suggest a marked weakening of l-NADW flow during the YD (Fig. 3e)^42^. Critically, within the range of age uncertainties, our $\overline{{{\rm{SS}}}}$ derived u-NADW strength is broadly anti-phased with the l-NADW during the deglaciation (Fig. 3a, b, d, e). The early weakening (~13.3 ka) and resumption (~12.3 ka) of the l-NADW before the onset and termination of YD (Fig. 3d, e) coincide with the strengthening and weakening of the u-NADW (Fig. 3a, b). This is consistent with previous work that shows opposite changes in Cd/Ca at intermediate^18^ and deep North Atlantic sites^17^ during the YD, suggesting a seesaw relationship between the u-NADW and l-NADW.

The inferred seesaw relationship between u-NADW and l-NADW can be explained by the coupled behaviour of the SPG and the Nordic Seas overflows during deglacial abrupt climate changes. This is supported by model simulations wherein a reduction in Nordic Seas deep convection (reducing l-NADW) alters ocean density gradients, which cause a strengthening of the SPG, that is sustained by positive feedback loops via salt and heat transports, resulting in increased isopycnal outcropping promoting an increase in deepwater formation in the subpolar North Atlantic (i.e., u-NADW)^43–47^. Additional modelling work^48^ also specifically highlights how a weakening of Iceland-Scotland Overflow Water – one of the two Nordic Seas overflows – reduces vertical density stratification, and thus enhances deep convection in the subpolar region, increasing u-NADW production.

### Surface-subsurface ocean warming: Northward Gulf Stream shift

Numerical model simulations suggest that the weakening of the l-NADW can also lead to a northward shift in the Gulf Stream^49^. This is because a key control on the position of the Gulf Stream is the strength of the northern recirculation gyre, which is formed due to vortex stretching and the conservation of potential vorticity as the Deep Western Boundary Current migrates downslope during its southward flow^32^. A reduction in the AMOC, particularly l-NADW^49^, is proposed to cause a northward shift in the Gulf Stream due to the reduction in the northern recirculation gyre. Paleoceanographic evidence from this region^11,50–53^ has often invoked this mechanism for recent centennial climate variability.

The Northwest Atlantic shelf-slope water system is situated on the fringe between the warm Gulf Stream and the cold Labrador Current (Fig. 1a), therefore, to determine the timing of northward shifts in the Gulf Stream during the last deglaciation, upper ocean and bottom water temperatures were reconstructed using planktonic foraminiferal assemblage counts and the planktonic and benthic foraminiferal Mg/Ca (Methods).

**Fig. 4:**
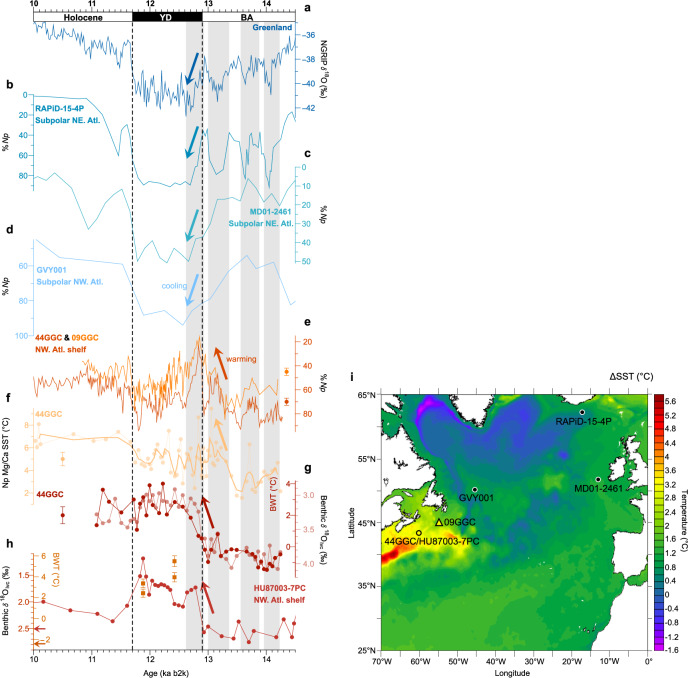
Distinct oceanic responses to abrupt deglacial climate change across the North Atlantic. **a** NGRIP *δ*^18^O^21^. Vertical grey shadings denote centennial-scale Greenland cold events. **b** RAPiD-15-4P %*Np*^55^. **c** MD01-2461 %*Np*^58^. **d** GVY001 %*Np*^57^. **e** %*Np* of 44GGC (red) and 09GGC (orange). **f** 44GGC *Np* Mg/Ca derived SST: raw data (line with dots) and 3pt-smoothed data (solid line). **g** 44GGC benthic *δ*^18^O_ivc_ (faint dark red) and Mg/Ca derived bottom water temperature (BWT, dark red) (based on *Globobulimina affinis*, *G. affinis*). In (**e**–**g**), error bars show averaged ± 1σ error. **h** HU87003-7PC benthic *δ*^18^O_ivc_ (red line with dots) and Mg/Ca derived BWT with 1σ error bars (orange squares) (based on *Cibicides lobatulus*, *C. lobatulus*). Late Holocene (0–4 ka) *δ*^18^O_ivc_ and BWT are indicated as red and orange arrows, respectively. **i** Map showing modelled North Atlantic ΔSST after a doubling of global atmospheric CO_2_ using the high-resolution global climate model CM2.6^5^ (colour shading) and locations of marine cores presented in panels (**b**–**h**) (RAPiD-15-4P, MD01-2461 and GVY001 are shown in black circles; 09GGC is shown in yellow triangle; 44GGC and HU87003-7PC are shown in yellow circle). The cooling or below-average warming (green-blue colour) and above-average warming (yellow-red colour) trends in the subpolar North Atlantic and Northwest Atlantic shelf are attributed to divergence in ocean heat transport and northward GS shift^5^, respectively. Note that the marine cores featured by typical Greenland-like cooling (**b**–**d**) at the YD onset fall within the cooling zone in (**i**), contrary to 44GGC, 09GGC and HU87003-7PC, which situate in the warming zone (**i**) and show pronounced surface-subsurface warming from BA to YD (**e**–**h**). Panel **i** adapted from ref. ^5^, with permission from Wiley.

The greenland climate and most of the subpolar North Atlantic surface ocean is characterised by an abrupt cooling at the onset of YD^21,54–58^ (Fig. 4a–d). In contrast, our high-resolution %*Np* (*Neogloboquadrina pachyderma*, Methods) records from the Northwest Atlantic – which we interpret as recording northward shifts in the location of the Gulf Stream – reveal an abrupt upper ocean warming at the onset of the YD (Fig. 4e). This warming occurs as part of a longer-term warming from ~13.5 to 12.8 ka, supported by similar trends in our *Np* Mg/Ca data (Fig. 4f). Moreover, a series of smaller amplitude centennial-scale warming events recorded in our %*Np* record oppose the centennial-scale cooling events in Greenland during the BA (Fig. 4a, e). Thus, our %*Np* data suggest that abrupt subpolar North Atlantic cold events were consistently associated with a northward shift of the Gulf Stream. This spatial pattern resembles the modern observed and modelled SST dipole (Fig. 4i)^5,8,9^.

The proposed northward migration of the Gulf Stream during the YD is further supported by a subsurface ocean warming recorded in benthic *δ*^18^O-Mg/Ca of 44GGC and HU87003-7PC, at 966 m and 920 m water depth, respectively (Fig. 4g, h). The 44GGC benthic *δ*^18^O exhibits a 0.80 ± 0.05 ‰ depletion during the YD, after correcting for global ice volume changes^59^ (*δ*^18^O_ivc_). This lighter benthic *δ*^18^O_ivc_ is primarily accounted for by a 3.0 ± 0.3 °C subsurface warming at intermediate depth (Fig. 4g), independently recorded in data from two benthic foraminifera species (Supplementary Fig. 6). Similarly, the HU87003-7PC benthic *δ*^18^O_ivc_ is 0.79 ± 0.07 ‰ and 0.77 ± 0.09 ‰ lower during the YD relative to the BA and late Holocene (Fig. 4h), respectively. As inferred from Mg/Ca data, this signal reflects a pronounced subsurface warming of 6.2 ± 0.7 °C in the YD relative to the late Holocene (Fig. 4h). The slightly larger temperature change as inferred from the Mg/Ca data recorded in the epifaunal species *C. lobatulus*, compared to our infaunal species at 44GGC (Fig. 4g), may be due to the additional influence of carbonate ion concentration on Mg/Ca in epifaunal species^60^. This interpretation is supported by elevated B/Ca^61^ and Sr/Ca^62^ ratios in these samples (Supplementary Fig. 8g, h), which are altered because of the changing bottom waters (i.e., more Gulf Stream-derived waters^63^) bathing the site. Previously reported subsurface warming during Heinrich Stadial events in the Northwest Atlantic has been attributed to the gradual diffuse spreading of subtropical water during intervals with a weakened AMOC^64–67^. However, a complete shutdown of intermediate circulation is invoked in the latter interpretation^65^, which clearly contradicts the observed strengthening of u-NADW during the YD (Fig. 3a–c). We therefore favour the interpretation that the subsurface warming recorded at our study location (i.e., the Laurentian and Scotian Slope region) during the YD is caused by the increased entrainment of Gulf Stream-derived water, especially given its proximal location to the Gulf Stream and observational evidence for the influence of Gulf Stream migrations on subsurface temperatures in this region^68^.

Several other mechanisms could also influence upper ocean temperature in the Northwest Atlantic shelf region, such as increased eddy heat flux, influence of along-slope advection, and basin-wide changes likely associated with external forcing. A weakening Labrador Current could in principle, drive warming at our core sites by reducing the advection of relatively cold water and enhancing local eddy activities^33^. However, this mechanism is inconsistent with a wide range of evidence showing a strengthened Labrador Current and u-NADW during the YD^18,34–40^ (Fig. 3a–c). Furthermore, the absence of upper ocean warming in the subtropical Northwest Atlantic^69^ precludes a basin-wide warming during the YD (Supplementary Fig. 9). Therefore, we favour the interpretation that the surface-subsurface warming in the Northwest Atlantic shelf region is driven by northward migration of the Gulf Stream. While our results point to a northward displacement and/or strengthening of the Gulf Stream influence in this region, future work is required to precisely constrain the finer details of Gulf Stream path geometry by increasing the spatial coverage of core materials from this region.

Our data, documenting a northward Gulf Stream shift contemporary with North Atlantic/Greenland cooling and a reduction in l-NADW, provides empirical support for the modelled response of the Gulf Stream to weakening l-NADW and its operation during past abrupt climate events of the deglaciation (Fig. 4i)^5,7–9,49^.

### Phasing of ocean reorganisation during the Younger Dryas onset and its implications

**Fig. 5:**
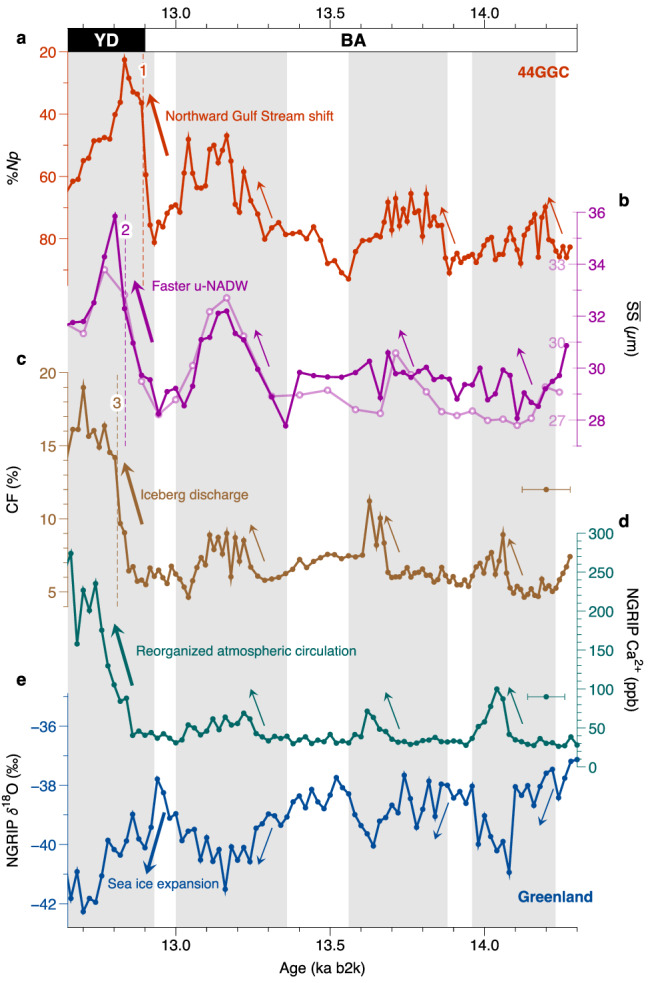
Co-ordinated ocean-atmospheric-ice response to abrupt deglacial climate change. **a**–**c** Downcore proxy records of 44GGC. **a** %*Np*. **b** $\overline{{{\rm{SS}}}}$ measured on Coulter (line with solid dots) and Malvern (line with open dots). **c** Percentage of coarse fraction (a proxy for IRD; Methods). Vertical dashed lines in (**a**–**c**) indicate the mean age of the mid-point for the BA-YD transition for each proxy derived from mid-point analysis (Methods). **d** NGRIP Ca^2+^^21^. **e** NGRIP *δ*^18^O^21^. Vertical grey shadings denote centennial-scale Greenland cold events as shown in Fig. 4. In (**c**, **d**), horizontal floating error bars denote average absolute age uncertainties ( ± 1σ)^88^.

Because we have a suite of proxy records constraining different features of North Atlantic circulation co-occurring in the same sediment core (44GGC), we are able to examine the relative phasing of events during the onset of the YD. The observed phasing is consistent with our inferred mechanism (Fig. 5a–c), but furthermore, it also supports the concept of an oceanic-leading mechanism during abrupt deglacial cooling events^41,70^. The clearest evidence is derived from the large magnitude changes at the onset of the YD, yet a similar sequencing is suggested for the earlier centennial cold events, albeit less clearly visible due to the smaller signal-to-noise ratio.

The earliest inferred change occurs in our *%Np* record (Fig. 5a), recording the northward shift of the Gulf Stream, in response to a weakening of l-NADW; we cannot, however, assess the precise phasing of this first relationship due to the proxy records deriving from different sediment cores. Following the initial l-NADW weakening and the connected northward Gulf Stream shift, we observe a 58 ± 38-year (95% CI) lagged increase in u-NADW, as inferred from the sharp increase in $\overline{{{\rm{SS}}}}$ (Fig. 5b). A similar duration lag has been reported in model experiments exploring the strengthening of the SPG and u-NADW in response to a weakening of l-NADW simulations, attributed to the delayed effect of the feedback mechanisms driving the change in u-NADW^44^. Critically, the distinct phase lag between our %*Np* and $\overline{{{\rm{SS}}}}$ records provides further support for the interpretation that the observed increase in $\overline{{{\rm{SS}}}}$ is not driven by changes in eddy activity. Such a mechanism would require a near-collapse of the Labrador Current^33^, which would instead lead to synchronous upper ocean warming (i.e., decrease in %*Np*) and enhanced eddy kinetic energy (i.e., increase in $\overline{{{\rm{SS}}}}$).

**Fig. 6:**
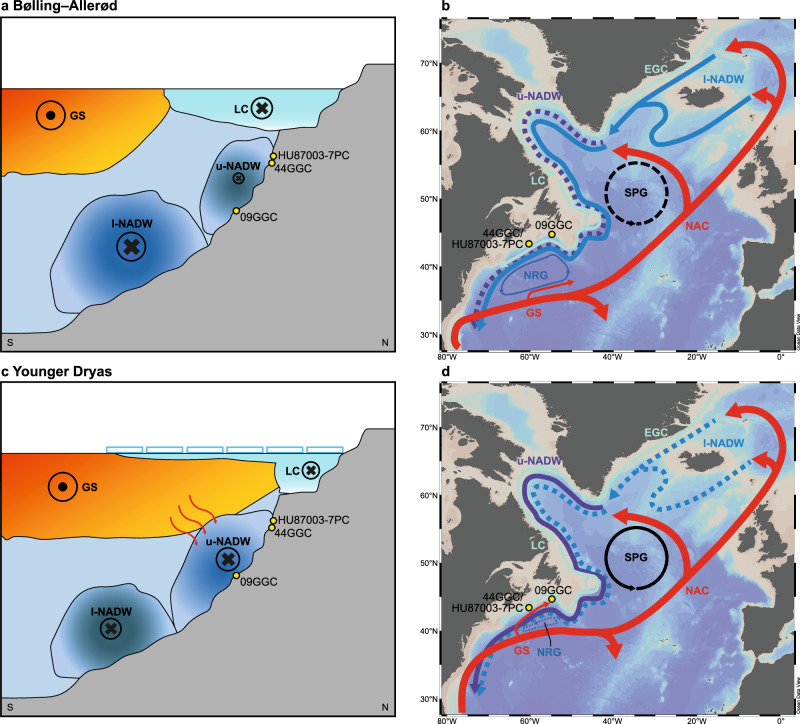
Schematic diagrams showing inferred changes in North Atlantic circulation during abrupt deglacial climate change. **a** The cross-section of the Northwest Atlantic shelf region during BA (Greenland warm intervals), where the surface ocean is likely dominated by cold, fresh Labrador Current and the subsurface is occupied by strong l-NADW and relatively weaker u-NADW. **b** Inferred North Atlantic circulation during BA (Greenland warm intervals), with a strong northern recirculation gyre, southernly positioned Gulf Stream, strong l-NADW, weak SPG and u-NADW. **c** As in (**a**) but for the scenario of YD, with a northward-shifted Gulf Stream that subducts underneath a freshwater cap in the surface. In the subsurface, the l-NADW and u-NADW are likely weakened and strengthened, respectively, with warm Gulf Stream-derived water entrained in the mid-depths. **d** As in (**b**) but for YD, with weakened northern recirculation gyre, northward shifted Gulf Stream, weakened l-NADW, strengthened SPG and u-NADW. GS Gulf Stream, LC Labrador Current, u/l-NADW upper/lower-North Atlantic Deep Water, NRG northern recirculation gyre, NAC North Atlantic current, SPG subpolar gyre, EGC East Greenland current. The line styles in (**b**, **d**) indicate relative strength, with solid and dashed/dotted lines reflecting stronger and weaker strengths, respectively. The maps in (**b**, **d**) were generated using Ocean Data View software^119^.

Finally, lagging the initial rapid ocean circulation change (i.e., the inferred northward Gulf Stream shift) by 84 ± 51 years (95% CI), there is a sharp increase in the delivery of icebergs (Fig. 5c), which, has been aligned with – and can be mechanistically coupled to – atmospheric circulation changes^22–26^, as inferred from ice core Ca^2+^ concentration^21^ (Fig. 2; Fig. 5d). This likely reflects the delayed adjustment of the atmosphere to the weakening of l-NADW and the subsequent shift in the SPG, and their cascading effects on basin-wide and inter-hemispheric ocean-atmosphere heat fluxes^71^. This ~100-year lag is consistent with the ~100-year lag observed between Greenland ice core *δ*^18^O and Ca^2+^ data (Fig. 5d, e), recording, respectively, the initial increase in subpolar sea-ice cover^72^ linked to the reduction of l-NADW, and the later hemispheric-wide atmosphere/climate reorganisation. Overall, this chain of events is consistent with a co-ordinated sequence of ocean circulation changes in the North Atlantic (Fig. 6), beginning with a decrease in l-NADW and a coupled shift in the Gulf Stream, which caused an increase in the SPG and u-NADW, culminating in the abrupt shift in atmospheric circulation.

This sequence of events, starting with l-NADW weakening, is consistent with the underlying mechanism for the YD (and earlier abrupt cold events) being associated with instability in Nordic Seas deepwater formation^73^, which tends to occur during intermediate climate states, such as the deglaciation^74^. Changes in Nordic Seas deepwater formation have commonly been attributed to internal ocean – sea-ice feedback processes within the Nordic Seas^75^, which were possibly modulated by the timing of Laurentide ice-sheet freshwater rerouting events, themselves linked to ocean-climate-ice sheet oscillatory behaviour^73^. The lagged response of u-NADW, and its stronger state during the YD, is inconsistent with the hypothesis that the trigger for the YD was a weakening of deepwater formation in the SPG (i.e., u-NADW).

### Surface-subsurface decoupling during the Younger Dryas

Our data show a coherent surface-subsurface ocean warming in the Northwest Atlantic in response to the proposed northward Gulf Stream shift preceding and during the YD onset (Fig. 4e–h). However, the surface-subsurface warming decouples during the YD, in which the subsurface keeps warming (Fig. 4g, h) while the surface ocean (*%Np* proxy data*;* and other published surface ocean proxies from the region^76,77^) returns to cold conditions after peak warmth at ~12.8 ka.

**Fig. 7:**
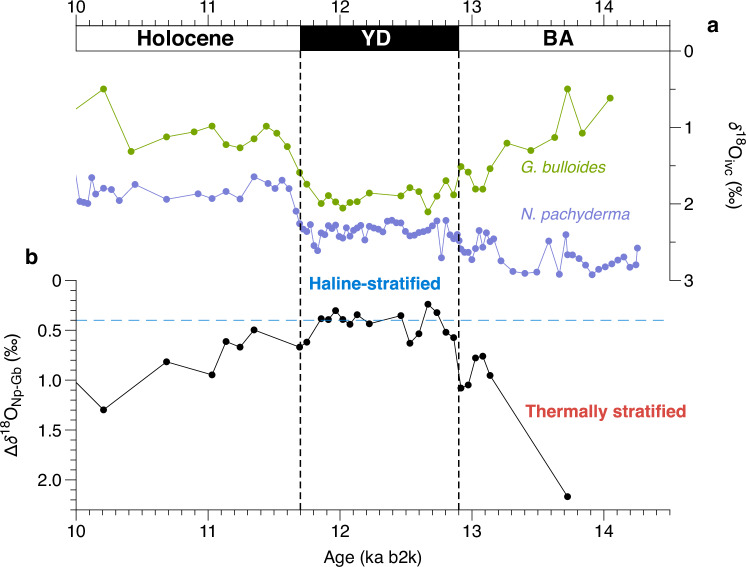
Surface ocean stratification regime reconstructed using Δδ^18^O_Np-Gb_ of 44GGC. **a** δ^18^O_ivc_ of planktonic foraminifera *Gb* (green) and *Np* (blue). **b** The offset between the δ^18^O of *Np* and *Gb* (Δδ^18^O_Np-Gb_), indicating stratification regimes of surface ocean, with < 0.4 ‰ values reflecting haline-stratified regime^78^.

This surface-subsurface decoupling is best explained by a change in the upper ocean stratification regime in the study area, as recorded by the difference in δ^18^O between the planktonic foraminifera species *Np* and *Globigerina bulloides* (*Gb*) (Δδ^18^O_Np-Gb_, “Methods”). During the BA, the large Δδ^18^O_Np-Gb_ values (> 0.75 ‰) imply a thermally stratified upper ocean in the Northwest Atlantic (Fig. 7); during the YD, when the Northwest Atlantic slope water system is affected by increased delivery of drifting icebergs/sea ice (Fig. 2), the Δδ^18^O_Np-Gb_ drops to ~0.4 ‰ (Fig. 7), indicating a haline-stratified regime^78^. In this regime, warm, saline Gulf Stream-derived water subducts below a fresh, sea ice-influenced surface layer, in a manner analogous to the western Nordic Seas today^78^. This interpretation is consistent with the *Np* Mg/Ca-derived SSTs, which typically resemble the %*Np* faunal data but show slightly warmer conditions in the YD than the BA (Fig. 4f) compared to the %*Np* record. This difference likely reflects suppressed heat loss to the atmosphere due to upper ocean stratification associated with a freshwater lid during the YD (Fig. 6c). Therefore, the decoupling between the surface and subsurface warming during the YD is likely associated with a haline-stratified upper ocean, when the Gulf Stream-derived water subducted below fresh, ice-bearing polar surface waters.

### Co-ordinated circulation changes in the past, present and future North Atlantic?

Our records suggest a strengthening of u-NADW and a northward shift of the Gulf Stream during the YD, which, based on the results of modelling studies, we suggest was an intrinsic, mechanistic response to a weakening of l-NADW^44,49,71^ (Figs. 5, 6). In addition to the onset of the YD, this relationship is also evident (albeit with more muted changes) during a series of abrupt centennial-scale Greenland cold events throughout the BA, revealing that this coupling of the Gulf Stream, u-NADW and l-NADW was a recurrent feature of these deglacial abrupt climate events. This, combined with its theoretical underpinning^32^, improves confidence in projections that a northward shift of the Gulf Stream may be expected under future scenarios in which l-NADW weakens^5^. Furthermore, because the northward migration of the Gulf Stream is a major factor affecting the health and spatial distribution of socio-economically important fisheries in the Northwest Atlantic region^79^, this finding is also of importance for the development of future marine ecosystem management plans.

Our results have relevance to understanding recent historical changes in the North Atlantic circulation. A prominent northward shift of the Gulf Stream occurred during the onset of the industrial era^11,53^, culminating in the Gulf Stream occupying its modern approximate location. At the same time, there has been an exceptional re-arrangement of the SPG circulation^80^, which models suggest was caused by a strengthening of the SPG^81^. Testing the hypothesis that these coordinated industrial era shifts in North Atlantic surface ocean circulation can be attributed to a concomitant weakening of l-NADW – in a manner analogous to the deglacial events we have described here – will require the development of robust historical records of l-NADW strength, as this interpretation remains to be directly tested, for which efforts are already underway.

## Methods

### Chronology and atmospheric link with Greenland ice core

The Radiocarbon-based age models for 44GGC and 09GGC were constructed based on ^14^C accelerator mass spectrometry (AMS) dates on planktonic foraminifera (Supplementary Table. 1) and the Vedde Ash tephra layer (Supplementary Fig. 2) identified within both marine cores and the NGRIP^21^. ^14^C ages were calibrated using the Marine20 calibration curve^82^ to account for variations in both atmospheric ^14^C concentration and global marine reservoir age, corrected with regional reservoir ages (ΔR) estimated from Vedde Ash tephra layer with uncertainties to account for variable ΔR throughout the deglaciation. Radiocarbon-based age models were thus constructed using Bayesian P_Sequence depositional models produced in OxCal v4.4^83^ with a variable k factor and individual Delta_R functions set for each date, to allow for time-varying offsets in sedimentation rate. A general outlier model (5%) was also specified for each depth except for records with the Vedde Ash tephra in, as there is a well-established independent age for this tephra^21^ (Supplementary Fig. 3).

However, the Marine20 curve must be applied cautiously to samples from polar regions during glacial/deglacial periods due to the spatially and temporally variable regional reservoir ages associated with sea ice, wind, and coastal upwelling^84,85^. Therefore, additional information is required to further improve the age models.

The Radiocarbon-based age model of 44GGC suggests significant increases in the percentage of coarse fraction (%CF) and IRD during the YD and intermittently in the BA (Fig. 2a), indicating an enhanced delivery of icebergs to the Northwest Atlantic, likely supplied from Baffin Bay and/or Hudson Strait and Labrador ice streams^86^. During the last glacial period, a series of millennial-scale IRD deposition events have been recorded in North Atlantic deep-sea sediments^22–26^, which are often closely linked with North Atlantic cold periods, when colder and dustier conditions are recorded in Greenland ice cores^87^. These IRD deposition events are synchronous across the North Atlantic and are attributed to contemporaneous iceberg discharges from various circum-North Atlantic ice sheets^22–26^. The near-synchronous iceberg calving from different glacial sources favours a climatic/atmospheric control rather than internal ice sheet dynamics, through mechanisms operating within the atmosphere that drive rapid oscillations in air temperature^22^ and/or moisture supplies^23^ to Northern Hemisphere high latitudes.

We therefore attribute the centennial- and millennial-scale increases in 44GGC IRD during BA and YD to increased iceberg calving in circum-North Atlantic ice sheets/ice streams. This interpretation is supported by the excellent correlation between our IRD record and NGRIP Ca^2+^ (a proxy for dust)^21^ (Fig. 2). Considering the age uncertainties associated with regional reservoir effects, we propose that the 44GGC IRD is synchronous with NGRIP Ca^2+^ via a fast atmospheric link. This correlation forms the basis for the Tuned age model of 44GGC (Fig. 2c, d). The age model was then transferred to 09GGC by correlating the percentage of polar planktonic foraminifera species *Np* while maintaining the tephra tie points (Fig. 2e, f), and to HU87003-7PC by correlating the *Np* δ^18^O (Supplementary Fig. 3a, e). Ages are reported in calendar dates before A.D. 2000 (b2k).

Notably, 44GGC (rather than 09GGC) was selected as the primary target for establishing the Tuned age model for two main reasons: (1) 44GGC is situated ideally for tracking Gulf Stream migration (Fig. 1a); (2) The more south-westerly position of 44GGC enables it to document a cleaner on-off signal for IRD deposition between cold and warm periods (Supplementary Fig. 4a, b) than compared to core 09GGC which is affected by more frequent drifting icebergs/sea ice and therefore exhibits lower signal-to-noise ratios (Supplementary Fig. 4d, e). Furthermore, the clearer IRD signal in 44GGC provides the basis for correlation with the NGRIP chronology (Fig. 2c, d).

Uncertainties of the Tuned age models were estimated using the P_Sequence depositional models: uncertainties within the tie-points to the NGRIP record were estimated visually, and chronological uncertainty errors were propagated with the overall uncertainty on the NGRIP records^88^. These were then input as a C_Date for the deeper portion of the record and supplemented with radiocarbon dates from the Holocene, where available, with a modern ΔR calculated from the deltar calculator^89^(see Supplementary Fig. 3).

The robustness of the Tuned age models is evaluated by estimating the regional reservoir ages using the stratigraphic age constraints. The calculated reservoir ages of the Northwest Atlantic are consistent with a previous study on high-latitude North Atlantic reservoir ages^27^, with a gradual increase through the BA to the YD before reaching peak values of 800–1000 ^14^C yr, followed by a decrease from the YD to early Holocene (Fig. 2g). In addition, the surface ^14^C reservoir ages of this study were also used for constraining the chronology of GGC36 based on published ^14^C dates^39^ (Fig. 2g and Supplementary Fig. 3d).

The robustness of the Tuned age model is further supported by the correlation between the NGRIP Ca^2+^ and 44GGC %CF (Fig. 2c, d and Supplementary Fig. 11). We compared the correlation between 44GGC %CF and NGRIP Ca^2+^ under the Radiocarbon-based and Tuned age models by interpolating both records onto a 20-year time step, which is plausible with respect to the resolution of both records. As expected, the correlation increases from *r* = 0.69 to *r* = 0.90 under the Tuned age model because the latter is aligned to the NGRIP record (Supplementary Fig. 11a–d). A Fisher r-to-z test confirms that this improvement is statistically significant; this was calculated using a coarser temporal resolution (100-year time step) to minimise sensitivity to the interpolation timestep (*z* = 2.06, *p *= 0.04). We then propagated chronological uncertainty using 10,000 Monte Carlo age perturbations, recalculating the correlation after randomly perturbing ages within their 1σ uncertainties (Supplementary Fig. 11e, f). Under the Radiocarbon-based age model, the correlation varies widely (median *r *= 0.66, 95% range 0.49–0.76), indicating that the inferred alignment is sensitive to associated age uncertainties. In contrast, the Tuned age model yields a consistently strong relationship (median *r* = 0.86, 95% range 0.81–0.89), and in all realisations the correlation exceeds that obtained under the Radiocarbon-based chronology. Taken together, this suggests that the Tuned age model is robust.

To summarise from a climatic perspective, our records indicate increased iceberg delivery to the Northwest Atlantic that are synchronous with cold conditions in Greenland, likely favoured by increased calving from glaciers feeding into the North Atlantic^22^. These intervals are accompanied by a rapid reorganisation in atmospheric circulation, leading to colder and dustier conditions in Greenland^87^, as well as a southward shift in polar front and expansion of seasonal sea ice in the subpolar North Atlantic^72^, which may further enhance iceberg delivery to the Northwest Atlantic by altering iceberg trajectories with shifted wind patterns^90^.

### Sample preparation

Cores 44GGC, 09GGC and HU87003-7PC were sampled every 1 cm throughout the deglaciation. Sediment samples were freeze-dried and weighed for dry bulk weight. Samples were then wet sieved using deionised water through 63 µm sieves to obtain the coarse ( > 63 µm) and fine fractions ( < 63 µm). The coarse fraction was subsequently dried at 40 ˚C and weighed, while the fine fraction was used for sortable silt grain size analysis.

### Sortable silt grain size analysis

The fine fraction samples of 44GGC and 09GGC were used for sortable silt (10–63 µm) grain size analysis. Samples were processed following published methods^30^ and analysed at University College London on a Beckman Coulter Multisizer 4 using the Enhanced Performance Multisizer 4 beaker and stirrer setting 30 for full sediment suspension. For each sample, two or three aliquots were analysed, with a sizing 70,000 particles per aliquot. Analytical error based on replicates from bulk sediment was ± 0.32 µm (*n* = 10). In addition, a subset of samples from each core was analysed on a Malvern Mastersizer 2000 to validate the current sorting effects in sediments delivered to the ocean, potentially derived from glacial sources following established protocol^91^. The $\overline{{{\rm{SS}}}}$ data are reported in arithmetic mean unless otherwise specified. Flow speed was quantified using established calibration^29^: Flow speed (m/s) = 1.2045 * $\overline{{SS}}$ − 20.12.

### Validation of sortable silt as a flow speed proxy

Previous studies suggest that sedimentary records located near glacial sources are likely affected by coarse-grained IRD, leading to the suspicion that the SS from those settings may be contaminated by unsorted glacial silt^92,93^. However, it has been argued that if the fine fraction has been transported and sorted, then it will provide a reliable flow history regardless its sources^91^. By examining the correlation between the SS mean ($\overline{{{\rm{SS}}}}$) and percentage (SS%), here we show that our SS records have been sufficiently well sorted and thus reflect near-bottom flow speed changes.

Strong positive correlations are found between $\overline{{{\rm{SS}}}}$ and SS% for both 44GGC and 09GGC (Supplementary Fig. 5a, b). This is consistent with the running downcore correlation between $\overline{{{\rm{SS}}}}$ and SS% (R_run_, Supplementary Fig. 5c, d), where the majority of records have passed the established validity test (R_run _> 0.5)^91^, demonstrating that the SS of the studied sites is controlled by current sorting processes. The brief time intervals where 44GGC fails the validity test (R_run _< 0.5) around mid-YD (~12.2 ka) and early Holocene (~10.8 ka), likely associated with more sluggish flows, do not coincide with the observed $\overline{{{\rm{SS}}}}$ peaks (Supplementary Fig. 5c).

SS records from cores on the slope to the west and north of Flemish Cap (1.5–2 km) have originally been suggested to be affected by IRD and thus corrected for IRD, leading to the interpretation that the intermediate flow is slower during the YD than BA^35^. However, their methods are contested given that the fine fraction of sediments in current-controlled deposits is unrelated to depositional mechanisms^36,91^.

### Cryptotephra scanning and geochemistry

Targeted intervals within the YD were selected for cryptotephra scanning. For 09GGC, the already washed foraminiferal > 150 µm fraction and the fine fraction of these samples were used, whereas for 44GGC sub-samples were sieved sequentially using 125 µm and 15 µm sieve meshes. Shards were then extracted from this fine fraction using a staged density separation method^94^ with amendments^95^, before samples were mounted in Canada balsam and counted using a high-powered polarising microscope. Peaks in tephra concentration were identified per gram of dry weight (Supplementary Fig. 2) and re-sampled for geochemical analysis. Individual grains were picked and encased in epoxy resin before being sectioned, polished and carbon-coated. Samples were analysed using the Cameca SX-100 electron microprobe analyser (EPMA) at the Tephra Analysis Unit in the University of Edinburgh. This uses a 3 µm beam and an accelerating 15 KeV voltage and a series of beam currents for different elements, ranging from 0.5 nA to 60 nA^96^. At the start and end of each analysis day, the internal standards Lipari and BCR-2G were run to ensure that there was no drift in the probe. The major elements from each sample were then compared to published tephra geochemistry and correlated to the Vedde Ash tephra^97^, which has been identified across the North Atlantic and Europe^98,99^, as well as located within the Greenland ice cores at 12,121 ± 57 b2k^100^.

### Paired foraminiferal δ^18^O-Mg/Ca analysis

Monospecific samples of planktonic foraminifera (*Np* and *Gb*) and benthic foraminifera (*G. affinis*, *C. lobatulus*, and *Nonionellina labradorica*, *N. labradorica*) were picked from narrow size ranges (typically 212–250 µm for planktonic and > 250 µm for benthic foraminifera, respectively) to limit ontogenic effects. Where sufficient material was available, approximately 50 planktonic and 15 benthic foraminiferal specimens were picked, gently crushed, homogenised and split for paired δ^18^O-Mg/Ca analysis, with ~35 % and ~65 % used for isotopic and trace metal analyses, respectively.

Stable isotope measurements of 44GGC were performed using a VG Isogas SIRA mass spectrometer with the MultiCarb preparation system (for sample size > 80 µg) and a Thermo MAT253 isotope ratio mass spectrometer with Kiel IV carbonate preparation device (for sample size < 80 µg) at the Godwin Laboratory, University of Cambridge, whilst those for HU87003-7PC were performed on a VG Prism instrument at NOSAMS, Woods Hole Oceanographic Institution. δ^18^O is reported relative to the VPDB standard, with analytical precision estimated to be better than ±0.07 ‰. The δ^18^O was corrected (δ^18^O_ivc_) to account for shifts in the global ocean caused by ice volume changes^59^.

The samples for trace metal analysis were cleaned using clay removal, reductive, oxidative, and weak acid leaching steps following established protocols^101–103^. Samples with > 5 µg post-cleaning weights were analysed for a suite of trace metal elemental ratios at the ICP-MS Trace Metal Lab at the University of Colorado, Boulder. *C. lobatulus* data were generated at Rutgers University. Long-term analytical precision for selected elements was 0.5% for Mg/Ca, 0.97% for Mn/Ca, 1.4% for Fe/Ca, and 0.8% for Al/Ca^104^.

Foraminiferal Mg/Ca may be significantly altered by post-depositional contamination by diagenetic^105^ and/or silicate phases^102^. Therefore, Mn/Ca, Fe/Ca and Al/Ca were used for screening against potential contamination based on their suggested threshold values (105, 100 and 400 µmol/mol for Mn/Ca, Fe/Ca and Al/Ca, respectively)^102,105,106^ and downcore covariances with Mg/Ca (Supplementary Figs. 7; Supplementary Fig. 8). For 44GGC, some diagnostic trace elemental ratios exceed the screening thresholds (e.g., Fe/Ca for *Np*; Mn/Ca and Fe/Ca for *G. affinis*; Mn/Ca and Fe/Ca for *N. labradorica*). However, the generally low covariance between these ratios and Mg/Ca after removing outliers ( > 2σ) indicates no systematic contamination due to insufficient cleaning (Supplementary Figs. 7, 8). For HU87003-7PC *C. lobatulus*, despite the measured Mn/Ca and Fe/Ca ratios covary with Mg/Ca (Supplementary Fig. 8i, j), their peak values are only marginally higher than the thresholds^105^, which could only account for < 0.1 °C ( < 1 %) additional warming in the YD when corrected for Mg/Ca^107^. We therefore conclude that temperature is the dominant control on the Mg/Ca variability in this study.

Screened Mg/Ca ratios were converted into SST and BWT for *Np*^108^, *G. affinis*^109^ and *C. lobatulus*^110^ using published species-specific calibrations. We note that *Np*-derived SSTs may represent an overestimated reconstruction, as previous work has shown that carbonate ion effects can exert a secondary control on *Np* Mg/Ca^111^. For *Np*, the combined analytical and calibration uncertainty is ±0.6 °C (1σ)^108^. For *G. affinis* and *C. lobatulus*, 1σ uncertainties were propagated by incorporating analytical error along with uncertainties in both the slope and intercept of the calibration equations^109,110^.

### Planktonic foraminifera assemblage

It should be noted that each planktonic foraminifera species has a specific depth habitat, thereby only recording the hydrographic conditions of the environments/water masses they dwell in refs. ^73,112^. We therefore performed planktonic foraminifera assemblage counts, which utilise the species’ preferences for different environments and thus provide a more reliable estimate for SST. The planktonic foraminifera assemblage is expressed as the percentage of the polar species *Np*, which is inversely correlated with SST^113^.

In this study, we performed *Np* counts on splits of the >150 µm size fraction with approximately 300 entities for each sample^114^, with the percentage of *Np* (%*Np*) determined by counting the number of *Np* against the total number of planktonic foraminifera present. ±1σ uncertainties were estimated using a binomial approach^115^.

### Ice-rafted detritus

During cold stadial events, massive discharges of icebergs are a ubiquitous feature in the subpolar North Atlantic, leaving a series of layers rich in IRD in deep sea sediments^116^. In this study, we counted the number of IRD grains > 150 µm per gram dry sediment. IRD was considered as the total number of lithogenic grains present.

### Surface ocean stratification regime

The difference in δ^18^O between the planktonic foraminifera species *Np* and *Gb* (Δδ^18^O_Np-Gb_; Fig. 7) is used to infer changes in stratification regime because the two species calcify at different water depths. The surface-dwelling *Gb* calcifies in the upper 60 m of the water column^117^, whereas the subsurface-dwelling *Np* inhabits a broad range of water depths, mostly well below the thermocline at 25–250 m^78^. This vertical habitat difference leads to relatively large Δδ^18^O_Np-Gb_ offsets ( > 0.4 ‰) in a thermally stratified regime with warmer water at the surface. In a haline-stratified regime, with the surface layer covered by freshwater/sea ice, however, planktonic foraminifera are forced to dwell deeper because they cannot tolerate low-salinity water, resulting in lower Δδ^18^O_Np-Gb_ ( < 0.4 ‰) analogous to western Nordic Seas today^78^.

### Mid-point analysis at the Younger Dryas onset

To determine the timing of the YD onset for each proxy record from core 44GGC (Fig. 5a–c), we defined the transition mid-point as the time at which the proxy reached the half-amplitude level, calculated as the midpoint between the maximum and minimum values within the interval 13.00–12.65 ka b2k. Because all proxy records are derived from the same sediment core and share a common age model (Supplementary Fig. 10), absolute age uncertainties are largely cancelled out when assessing relative phasing. We therefore propagated relative chronological uncertainty between tie points by applying 10,000 bootstrap realisations of stochastic linear stretching and compression of the transition window (13.00–12.65 ka b2k), constrained by ± 1σ age uncertainties at the bounding ages. For each realisation, the proxy records were linearly interpolated onto a uniform 1-year time step, and the mid-point age was recalculated. Lead-lag distributions were then derived from the ensemble of warped time axes.

## Supplementary information

Supplementary Information

Transparent Peer Review file

## Source data

Source Data

## Data Availability

All data generated for this study have been made available through the data repository Pangaea at https://doi.pangaea.de/10.1594/PANGAEA.995150. Source data are provided in this paper.

## Code availability

Python and R code used to perform statistical analyses as part of this study are available on Zenodo at 10.5281/zenodo.20401358.
