# Supplementary material for: Co-ordinated shifts in deep-water formation and Gulf Stream migration during abrupt climate changes
Source: Nat Commun. 2026 Jun 11;17:4966. doi: 10.1038/s41467-026-73832-4 (PMC13260390; doi:10.1038/s41467-026-73832-4)
Supplement: Supplementary file 1 — Supplementary Information [file 41467_2026_73832_MOESM1_ESM.pdf]

Supplementary Information

Co-ordinated Shifts in Deep-water Formation and Gulf Stream Migration During Abrupt Climate Changes

Fangjingcheng Zhu[1*†], Alice Carter-Champion[1,2], Jack H. Wharton[1], Joel Bracamontes-Ramírez[3], Andrea Burke[4], Peter B. de Menocal[5], David Fairman[1], Lloyd D. Keigwin[5], Thomas M. Marchitto[6], Eirini Papachristopoulou[1], James W. B. Rae[4], Yair Rosenthal[7,8], Ning Zhao[9], David J. R. Thornalley[1,5*]

[1]Department of Geography, University College London, London, UK.

[2]Centre for Quaternary Research, Department of Geography, Royal Holloway, University of London, London, UK

[3]Department of Earth System Sciences, University of Hamburg, Hamburg, Germany

[4]School of Earth and Environmental Sciences, University of St Andrews, St Andrews, UK

[5]Woods Hole Oceanographic Institution, Woods Hole, MA, USA

[6]Department of Geological Sciences and INSTAAR, University of Colorado, Boulder, CO, USA

[7]Department of Marine and Coastal Sciences, Rutgers University, New Brunswick, NJ, USA

[8]Department of Earth and Planetary Sciences, Rutgers University, New Brunswick, NJ, USA

[9]State Key Laboratory of Estuarine and Coastal Research and School of Marine Sciences, East China Normal University, Shanghai, China

[*]Corresponding authors: fangjingcheng.zhu.20@alumni.ucl.ac.uk; d.thornalley@ucl.ac.uk

[†]Present address: School of Ocean and Earth Science, University of Southampton, Waterfront Campus, National Oceanography Centre, Southampton, UK

Supplementary Table 1. The ^{14}C AMS dates and surface reservoir ages. (* Data were omitted due to age reversals.)

Site	Depth (cm)	Planktic foraminifera	^{14}C age (years B.P.)	± (years)	Tuned age (ka b2k)	R_{surf} (years)
44GGC	139.75	Mixed planktic	9055	30	10.09	216
44GGC	167.75	Mixed planktic	9970	70	10.92	421
44GGC	204.25	Mixed planktic	10185	40	11.41	250
44GGC	224.25	Mixed planktic	10650	70	11.67	636
44GGC	235.75	*Np*	10715	50	11.83	624
44GGC	266.75	*Np*	11150	60	12.27	802
44GGC	304.75	*Np*	11590	40	12.90	651
44GGC	352.75	*Np*	12390	70	13.72	596
44GGC	396.75	*Np*	12660	60	14.25	405
09GGC	200.25	*Gb*	9890	40	10.83	390
09GGC	216.25	*Gb*	10100	40	11.05	594
09GGC	242	*Np*	10870	60	11.74	823
09GGC	262.25	*Gb*	10960	210	11.97	796
09GGC	283.75	*Np*	11150	35	12.17	841
09GGC	284.75	*Gb*	11210	40	12.18	905
09GGC	308.25	*Np*	11225	50	12.26	881
09GGC	319.75	*Np*	11375	30	12.32	1004
09GGC	372.75	*Np*	11635	45	12.82	792
09GGC	399.25	*Np*	11865	40	13.05	863
09GGC	424.25	*Np*	12215	40	13.26	918
09GGC	452.25	*Np*	12455	40	13.40	999
09GGC	484.25	*Np*	12460	60	13.55	854
09GGC	496.25	*Np*	13515*	40	13.63	1874*
09GGC	516.25	*Np*	13310*	70	13.76	1536*
09GGC	536.25	*Np*	12745	35	13.90	782
09GGC	551.25	*Np*	12855	35	14.20	644
HU87003-7PC	282	*Gb*	10100	70	11.10	604
HU87003-7PC	402	*Gb*	11350	65	12.78	644

Supplementary Table 2. Site locations

Site	Latitude	Longitude	Water Depth	Reference
44GGC	43°21.01' N	60°12.48' W	966 m	This study
09GGC	44°49.60' N	54°53.78' W	1854 m	This study
HU87003-7PC	43°20.70' N	60°12.90' W	920 m	This study
GGC36	43°05.94' N	49°02.00' W	1520 m	Zhao et al. (2018)[1]
GVY001	50°09.60' N	45°30.60' W	3721 m	Zhou et al. (2021)[2]
MD01-2461	51°45' N	12°55' W	1153 m	Peck et al. (2008)[3]
TTR-451	58°30.89' N	44°54.33' W	1927 m	Stanford et al. (2006)[4]
RAPiD-15-4P	62°17.58' N	17°08.04' W	2133 m	Thornalley et al. (2010)[5]

MD99-2284	62°22.48’ N	0°58.81’ W	1500 m	Muschitiello et al. (2019)[6]
NGRIP	75°05’ N	42°17’ W	-	NGRIP members (2004)[7]

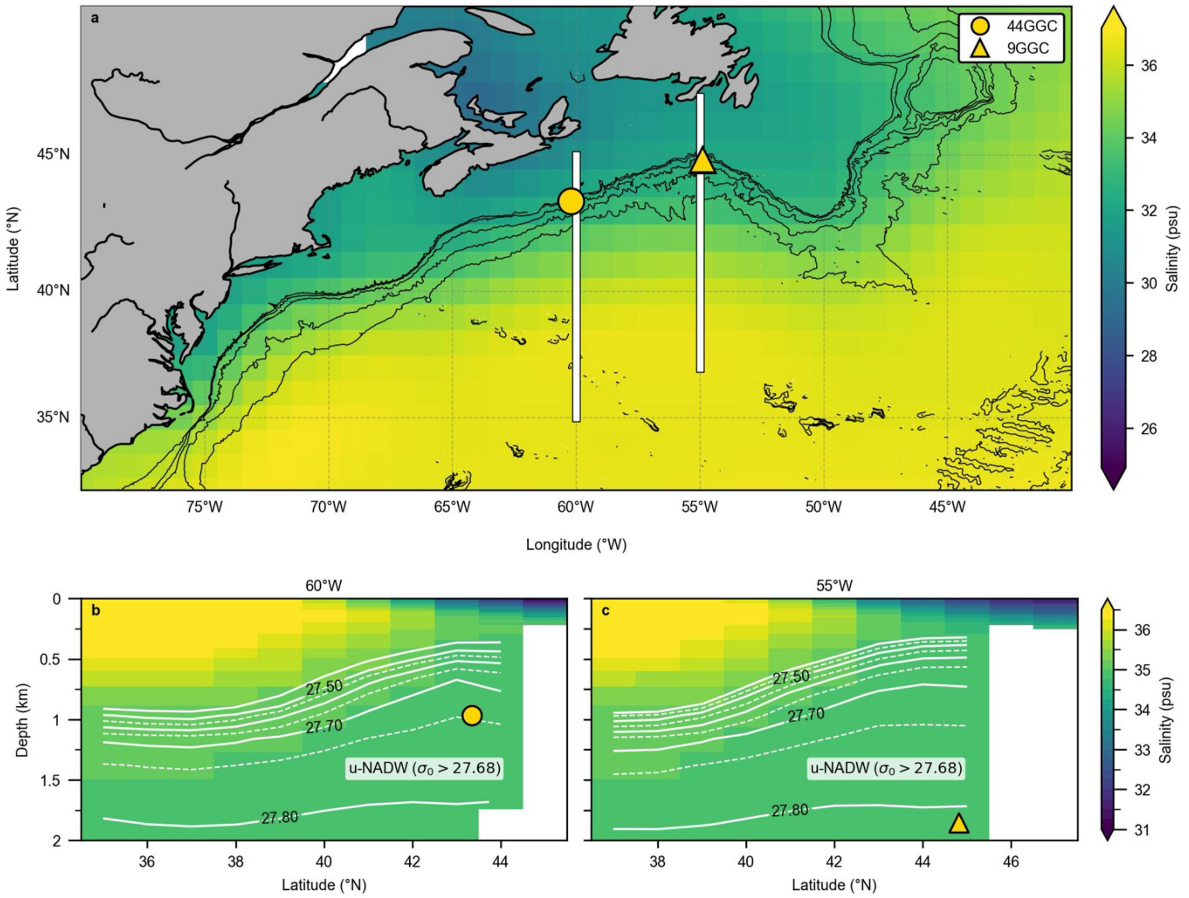

Supplementary Fig 1. Salinity (colour shading) and potential density (σ_0, kg m^{-3}; white contours) in the northwest Atlantic derived from EN4.2.1, averaged between January 2000 and January 2020. **a** Sea-surface salinity with bathymetric contours at 0.5, 1, 2, 3, and 4 km water depth (black contours). Ocean bathymetry is from the GEBCO_2014 global bathymetric grid (30 arc-second resolution)[22]. **b–c** Salinity and potential density sections at 60°W (**b**) and 55°W (**c**). Solid white lines in **a** indicate the locations of the zonal sections shown in **b** and **c**. Yellow symbols mark the locations of the sediment core sites, both of which are situated at depths currently bathed by waters with densities characteristic of upper North Atlantic Deep Water (u-NADW; $\sigma_0 > 27.78$ kg m^{-3})[8] (Note that density value associated with 09GGC is marginally high due to the coarse resolution of the EN4 dataset.).This indicates that the sites are influenced by u-NADW rather than deeply penetrating slope waters, implying that the sortable-silt mean grain-size reconstructions are representative of u-NADW flow speeds. EN4 provides monthly, gridded objective analyses of available ocean observations at 1° horizontal resolution[9]; fields and sections were extracted by time-averaging salinity and temperature for the 2000–2020 period. Density was computed using the Python implementation of the Gibbs SeaWater Oceanographic Toolbox[10].

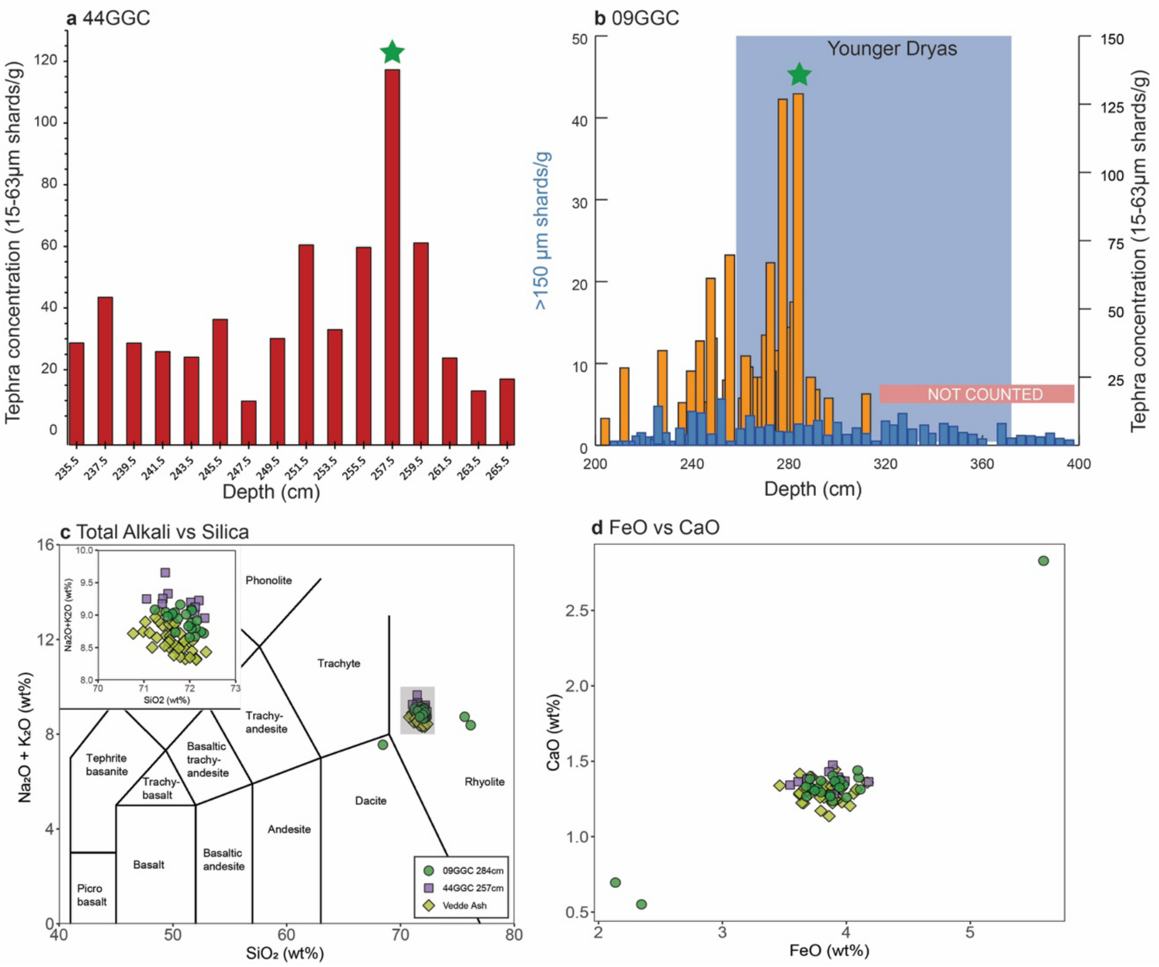

Supplementary Fig 2. Summary of cryptotephra peaks found in the Vedde Ash targeted interval for a 44GGC and b 09GGC, based on preliminary radiocarbon dates. The peak at 257–258cm (equivalent to 260.25–261.25cm offset depth) in 44GGC is assigned as the isochron, whereas the highest shard concentration in 09GGC is located at 283.5–284cm. These peaks were resampled and geochemically analysed. **c** Total alkali vs silica plot[11] of the peaks in 44GGC and 09GGC in comparison to the published Vedde Ash chemistry from Krakenes lake in Norway[12], highlighting the coherence of these peaks with the published Vedde Ash. **d** Additional biplot of FeO and CaO highlighting the correlation of 44GGC and 09GGC to the Vedde Ash.

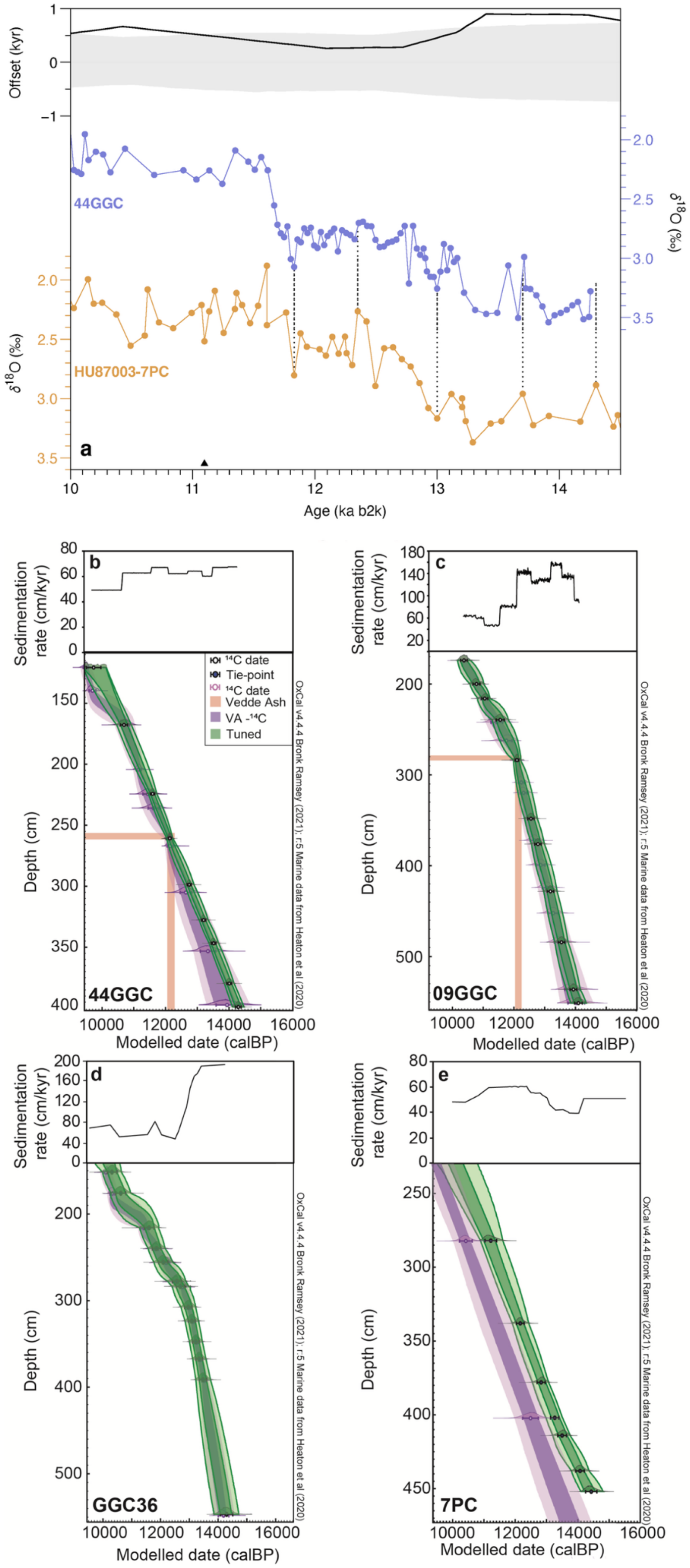

Offset (kyr)
44GGC
HU87003-7PC
δ18O (‰)
Age (ka b2k)
a
b
c
d
e
Sedimentation rate (cm/kyr)
Depth (cm)
Modelled date (calBP)
14C date
Tie-point
14C date
Vedde Ash
VA -14C
Tuned
44GGC
09GGC
GGC36
7PC
OxCal v4.4.4 Bronk Ramsey (2021); r:5 Marine data from Heaton et al (2020)

Supplementary Fig 3. Chronology and age-depth relationship. **a** Upper: Temporal offset between the Radiocarbon-based and Tuned age models of HU87003-7PC (black line). Also shown is the uncertainty range of the Radiocarbon-based age model (grey shading; 95% confidence interval, CI). Lower: *Np* $\delta^{18}O$ of 44GGC (blue) and HU87003-7PC (yellow). The age model of HU87003-7PC was based on correlation of *Np* $\delta^{18}O$ with 44GGC (dotted lines) and a ^{14}C date calibrated using Marine20[13] (black triangle). **b** Upper: Sedimentation rate of 44GGC. Lower: Depth vs age of 44GGC. Dark purple and dark green shadings denote 68% confidence intervals of the Radiocarbon-based and Tuned age models, respectively. Pale purple and pale green shadings denote 95% confidence intervals of the Radiocarbon-based and Tuned age models, respectively. Calibrated ^{14}C dates used in the Radiocarbon-based age model are shown in purple circles with error bars denoting ±1σ error. Stratigraphic tie points (filled black circles) and calibrated ^{14}C dates (open black circles) used in the Tuned age model are shown with error bars denoting ±1σ error. The pale red shadings denote the Vedde Ash tephra layer with ±2σ error[14]. **c–e** As in **(b)** but for 09GGC, GGC36, and HU87003-7PC, respectively.

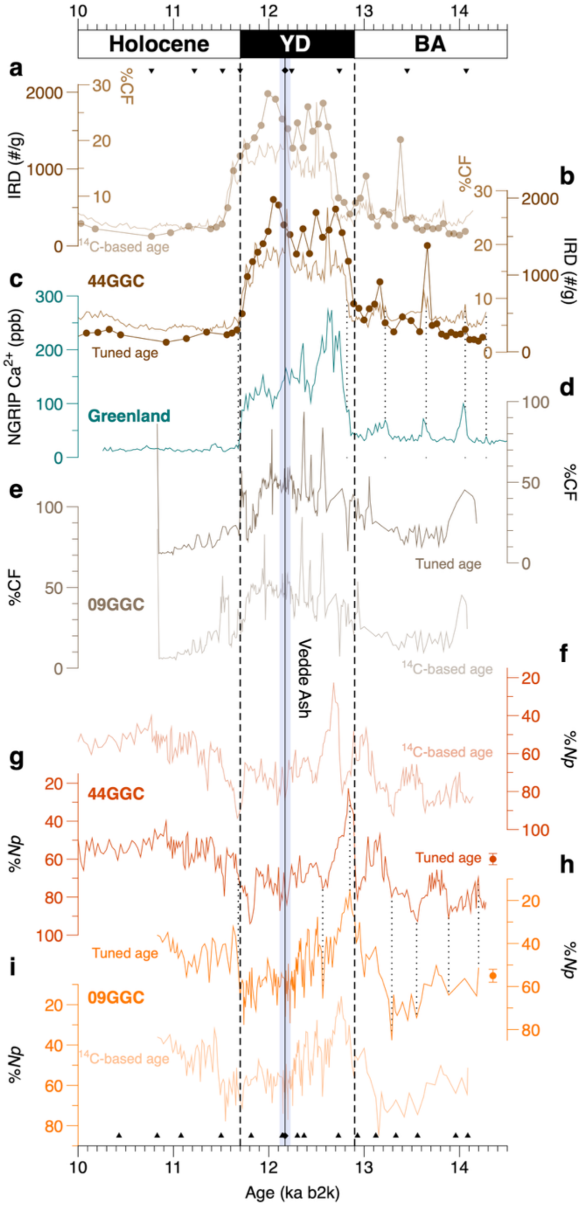

Supplementary Fig 4. Radiocarbon-based and Tuned age models of 44GGC and 09GGC. **a** 44GGC %CF (light brown line) and IRD (dark brown line with dots) plotted on the

Radiocarbon-based age model based on a tephra tie point (diamond) and ^{14}C dates (inverted triangles) calibrated using the Marine20 calibration curve[13], corrected with regional reservoir age (ΔR) estimated from Vedde Ash tephra layer with uncertainties to account for variable ΔR throughout the deglaciation. **b** Same as in (**a**) but plotted on the Tuned age model using the tephra tie point (vertical solid line) and the correlation of %CF and IRD with NGRIP Ca^{2+} (tie points indicated by dotted lines). **c** NGRIP Ca^{2+}[14]. **d** 09GGC %CF plotted on the Tuned age model. **e** 09GGC %CF plotted on the Radiocarbon-based age model. **f** 44GGC %*Np* plotted on the Radiocarbon-based age model. **g** 44GGC %*Np* plotted on the Tuned age model. **h** 09GGC %*Np* plotted on the Tuned age model synchronized with 44GGC using the tephra tie point (vertical solid line) and correlation with 44GGC %*Np* (tie points indicated by dotted lines). The error bars in **g** and **h** denote averaged ±1σ errors. **i** 09GGC %Np plotted on the Radiocarbon-based age model based on a tephra tie point (diamond) and ^{14}C dates (triangles) calibrated using the Marine20 calibration curve[13], corrected with ΔR estimated from Vedde Ash tephra layer with uncertainties to account for variable ΔR throughout the deglaciation. The age of the Vedde Ash tephra layer is indicated in a solid vertical line with shading denoting ±1σ error[14].

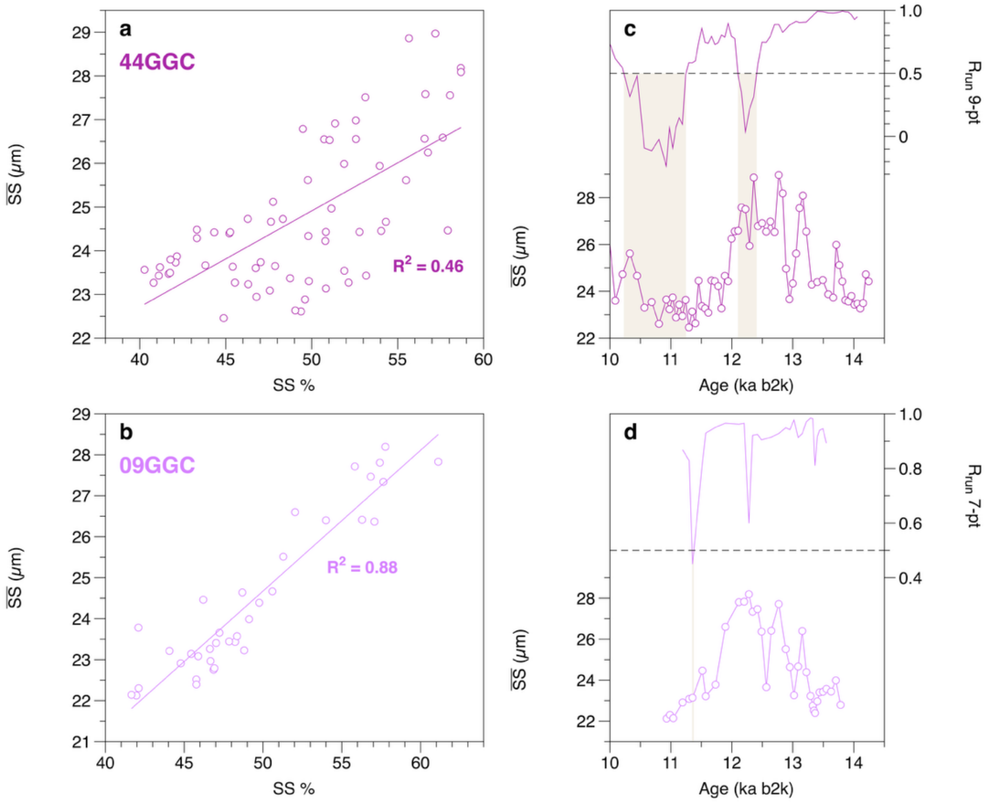

Supplementary Fig 5. $\overline{SS}$ vs SS%, and R_{run} for validating SS as a flow speed proxy. $\overline{SS}$ vs SS% for (**a**) 44GGC and (**b**) 09GGC. Running downcore correlation between $\overline{SS}$ and SS% (R_{run}) plotted along with $\overline{SS}$ for (**c**) 44GGC and (**d**) 09GGC. 9-point and 7-point smoothed R_{run} are used for 44GGC and 09GGC, respectively. Light beige shadings denote intervals where $\overline{SS}$ data do not pass the validity test (R_{run} < 0.5)[15], indicating poor sorting and unreliable records. SS data were measured from Malvern. Note $\overline{SS}$ data are presented as geometric mean for the validity test shown here, following the established method[15].

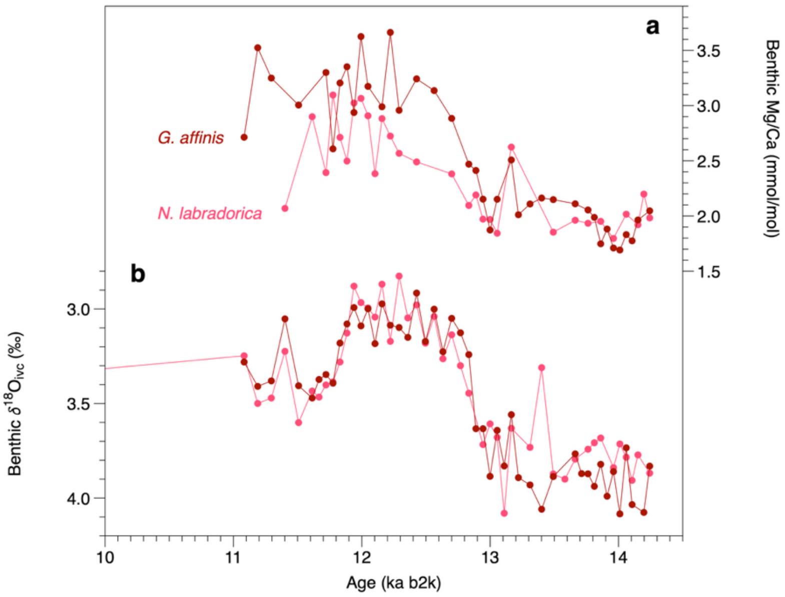

Supplementary Fig 6. Comparison of Mg/Ca (a) and $\delta^{18}O_{ivc}$ (b) between benthic foraminifera *G. affinis* (dark red) and *N. labradorica* (pink) of 44GGC.

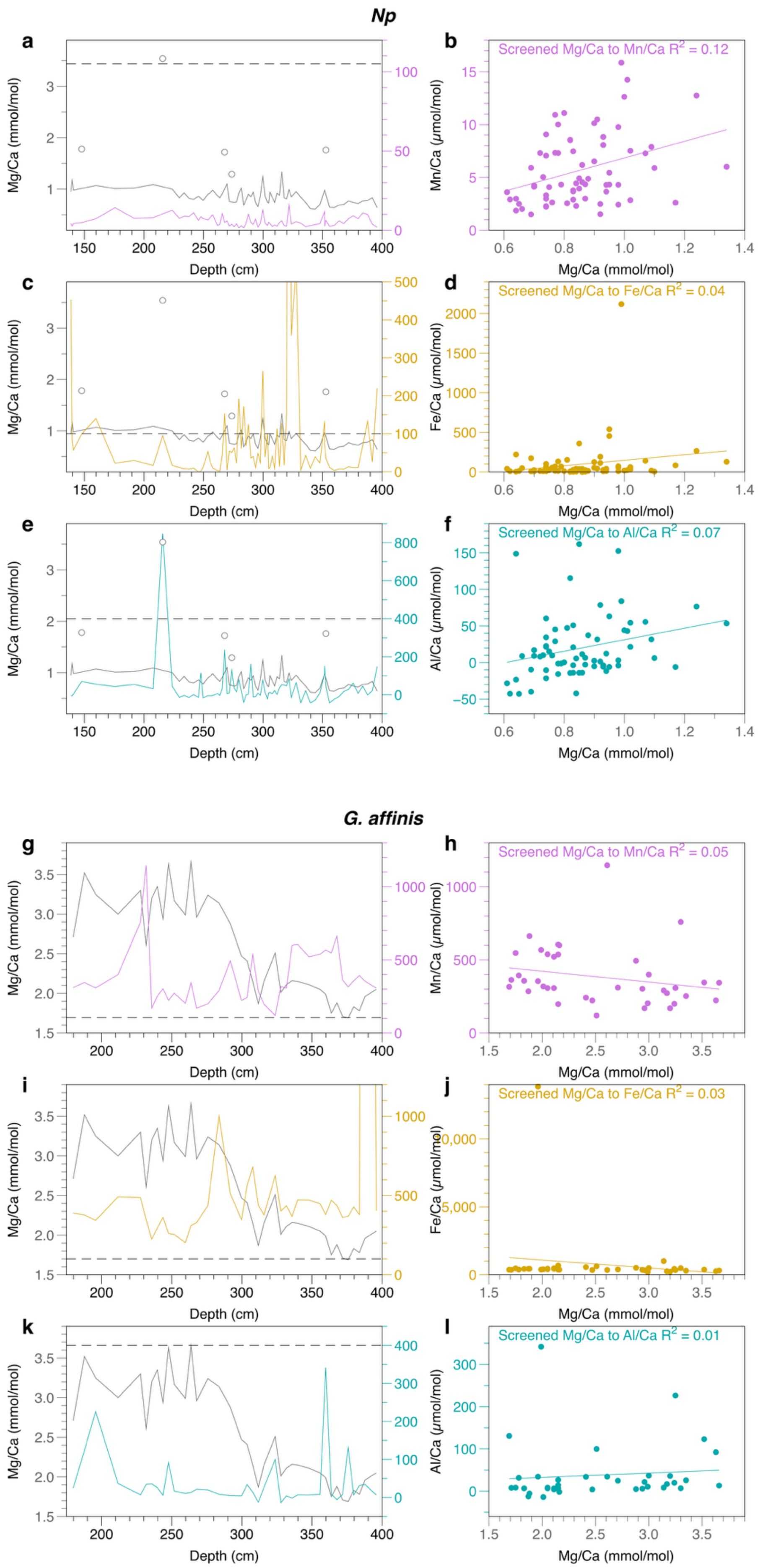
Np
G. affinis
a
b
c
d
e
f
g
h
i
j
k
l
Mg/Ca (mmol/mol)
Depth (cm)
Mn/Ca (µmol/mol)
Fe/Ca (µmol/mol)
Al/Ca (µmol/mol)
Screened Mg/Ca to Mn/Ca R2 = 0.12
Screened Mg/Ca to Fe/Ca R2 = 0.04
Screened Mg/Ca to Al/Ca R2 = 0.07
Screened Mg/Ca to Mn/Ca R2 = 0.05
Screened Mg/Ca to Fe/Ca R2 = 0.03
Screened Mg/Ca to Al/Ca R2 = 0.01

Supplementary Fig 7. Trace element content of 44GGC *Np* (a–f) and *G. affinis* (g–l). Downcore contaminants (**a, c, e, g, i, k**) of Mn/Ca (purple), Fe/Ca (gold), and Al/Ca (turquoise) plotted against Mg/Ca (light grey) with general contamination limit for each element[16–18] (dashed lines). Rejected data points are shown in open circles. Cross plot of screened Mg/Ca against each contaminant (**b, d, f, h, j, l**) indicate no covariance between these trace metals.

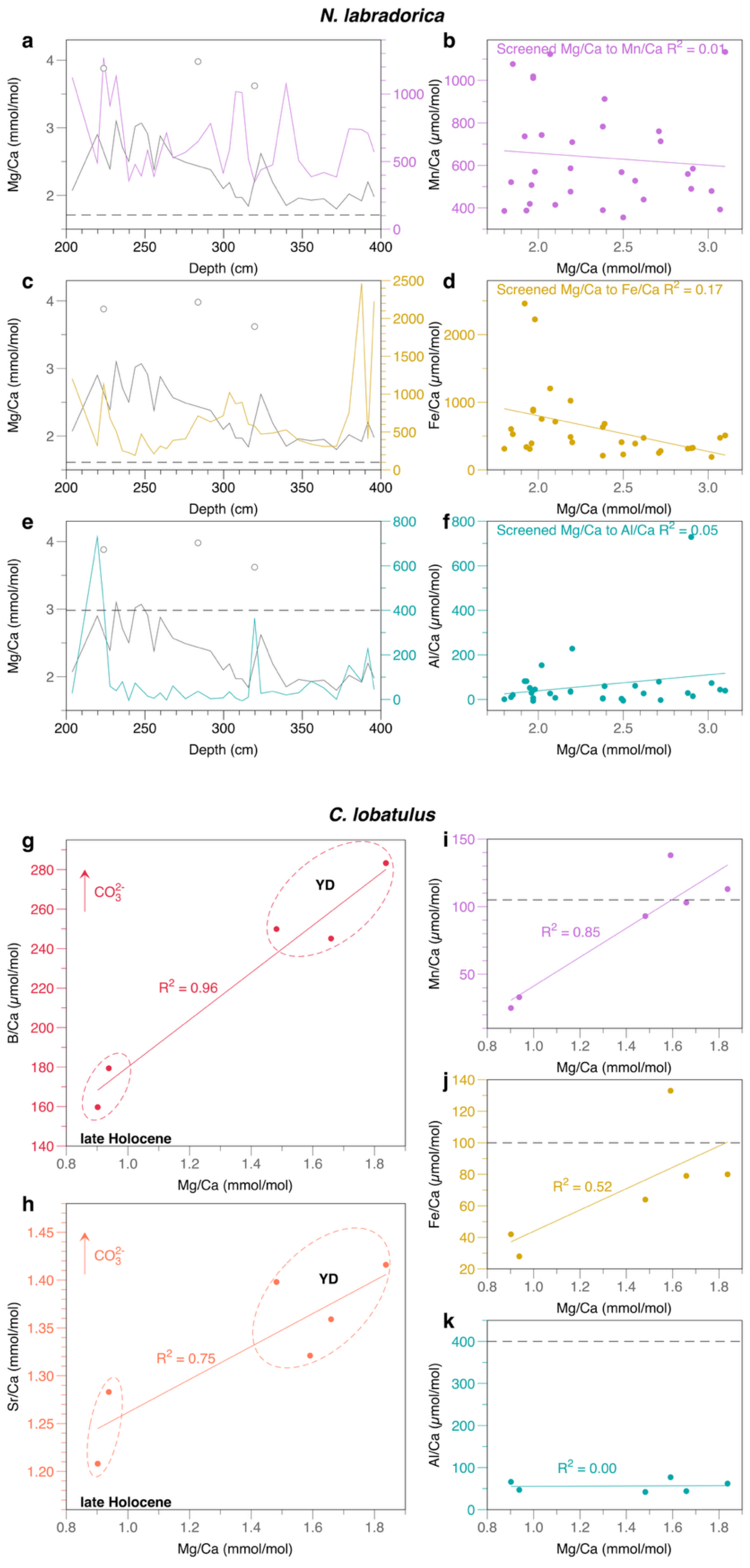

N. labradorica
a
b
c
d
e
f
Mg/Ca (mmol/mol)
Depth (cm)
Mn/Ca (µmol/mol)
Fe/Ca (µmol/mol)
Al/Ca (µmol/mol)
Screened Mg/Ca to Mn/Ca R2 = 0.01
Screened Mg/Ca to Fe/Ca R2 = 0.17
Screened Mg/Ca to Al/Ca R2 = 0.05
C. lobatulus
g
h
i
j
k
B/Ca (µmol/mol)
Sr/Ca (mmol/mol)
CO3 2-
YD
late Holocene
R2 = 0.96
R2 = 0.75
R2 = 0.85
R2 = 0.52
R2 = 0.00

Supplementary Fig 8. Trace element content of 44GGC *N. labradorica* (a–f) and HU87003-7PC *C. lobatulus* (g–k). Downcore contaminants (**a, c, e**) of Mn/Ca (purple), Fe/Ca (gold), and Al/Ca (turquoise) plotted against Mg/Ca (light grey) with general contamination limit for each element[16–18] (dashed lines). Rejected data points are shown in open circles. Cross plot of screened Mg/Ca of 44GGC *N. labradorica* against each contaminant (**b, d, f**) indicate no covariance between these trace metals. Cross plots of Mg/Ca of HU87003-7PC *C. lobtatulus* against **g** B/Ca and **h** Sr/Ca suggest higher carbonate ion during the YD[19,20]. Also shown are cross plots of Mg/Ca of HU87003-7PC *C. lobtatulus* against each contaminant (**i–k**) with general contamination limit for each element [16–18] (dashed lines), indicating contaminant concentration either lower (**k**) or only marginally higher than the limit (**i, j**).

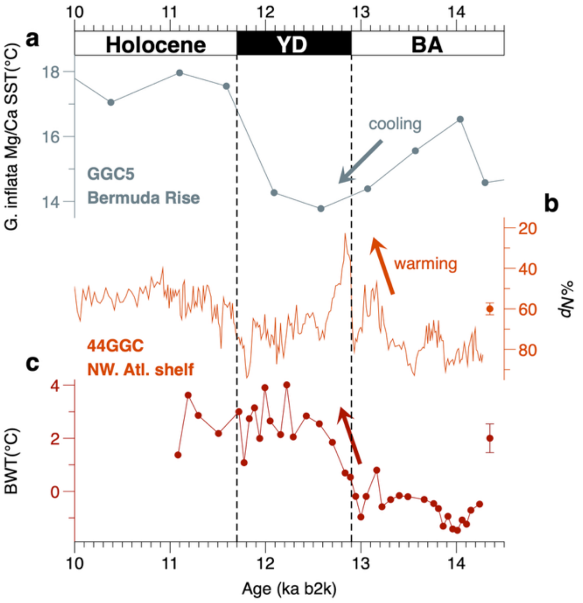

Supplementary Fig 9. Comparison of ocean temperature records between Northwest Atlantic shelf and subtropical Northwest Atlantic. **a** *G. inflata* Mg/Ca derived SST[21] of GGC5 from Bermuda Rise in the subtropical Northwest Atlantic. **b** %*Np* of 44GGC (this study). **c** 44GGC benthic Mg/Ca derived bottom water temperature (BWT). In **b** and **c**, error bars show averaged ±1σ error.

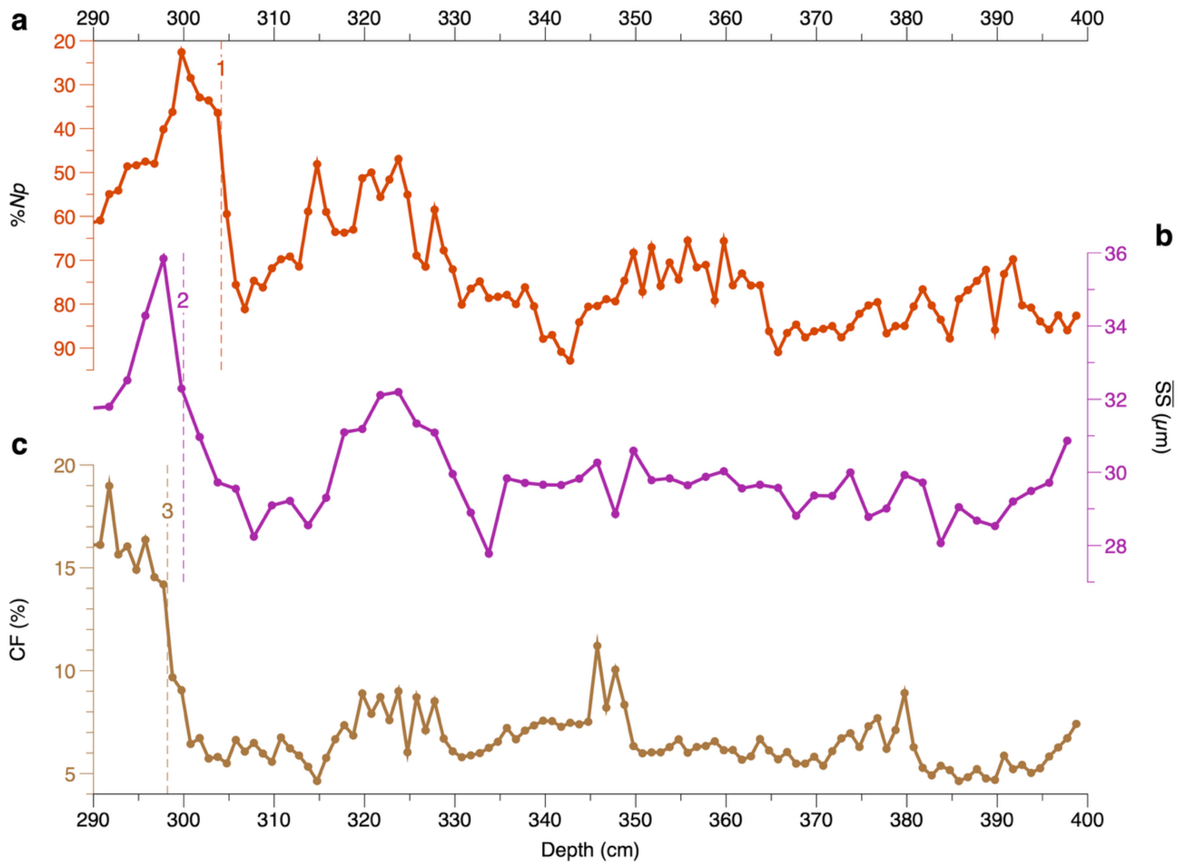

Supplementary Fig 10. Mid-point analysis at the YD onset (data plotted on depth). a %*Np*. **b** $\overline{\mathrm{SS}}$ measured on Coulter. **c** Percentage of coarse fraction (a proxy for IRD; Methods). Vertical dashed lines in **a**–**c** indicate the mean depth of the mid-point for BA-YD transition for each proxy derived from bootstrapping analysis (Methods).

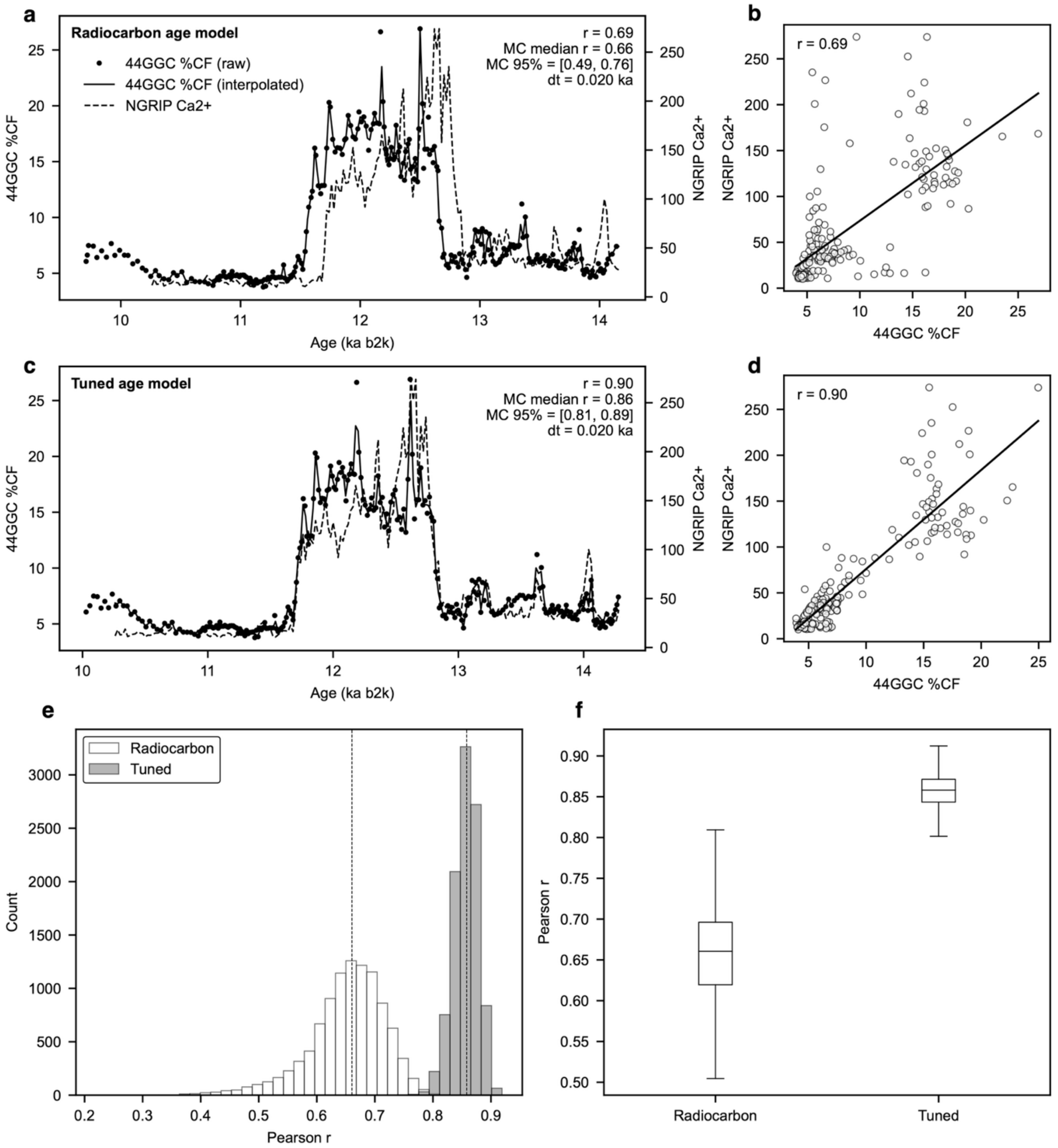

Supplementary Fig 11. Correlation analysis for NGRIP Ca^{2+} vs 44GGC %CF under the Radiocarbon-based and Tuned age models. a Time series of NGRIP Ca^{2+} (dashed line)[14] and 44GGC %CF under the Radiocarbon-based age model (raw data: dots; interpolated data: solid line). **b** Scatter plot for interpolated NGRIP Ca^{2+} and 44GGC %CF under the Radiocarbon-based age model. **c** As in **a** but for the Tuned age model. **d** As in **b** but for the Tuned age model. **e** Monte Carlo distributions of correlation coefficients (r) between NGRIP Ca^{2+} and 44GGC %CF under the Radiocarbon-based (white) and Tuned (grey) age models, obtained by randomly perturbing ages within 1σ uncertainties. **f** Box-and-whisker representation of the same Monte Carlo correlation distributions shown in **e**, for the Radiocarbon-based (left) and Tuned (right) age models. Centre lines indicate medians; boxes indicate the interquartile range; whiskers indicate 1.5× the interquartile range.

References

1. Zhao, N., Marchal, O., Keigwin, L., Amrhein, D. & Gebbie, G. A Synthesis of Deglacial Deep-Sea Radiocarbon Records and Their (In)Consistency With Modern Ocean Ventilation. *Paleoceanogr. Paleoclimatol.* **33**, 128–151 (2018).

2. Zhou, Y. *et al.* Enhanced iceberg discharge in the western North Atlantic during all Heinrich events of the last glaciation. *Earth Planet. Sci. Lett.* **564**, 116910 (2021).

3. Peck, V. L., Hall, I. R., Zahn, R. & Elderfield, H. Millennial-scale surface and subsurface paleothermometry from the northeast Atlantic, 55-8 ka BP. *Paleoceanography* **23**, PA3221 (2008).

4. Stanford, J. D. *et al.* Timing of meltwater pulse 1a and climate responses to meltwater injections. *Paleoceanography* **21**, PA4103 (2006).

5. Thornalley, D. J. R., McCave, I. N. & Elderfield, H. Freshwater input and abrupt deglacial climate change in the North Atlantic. *Paleoceanography* **25**, PA1201 (2010).

6. Muschitiello, F. *et al.* Deep-water circulation changes lead North Atlantic climate during deglaciation. *Nat. Commun.* **10**, 1272 (2019).

7. North Greenland Ice Core Project (NGRIP) members. High-resolution record of Northern Hemisphere climate extending into the last interglacial period. *Nature* **431**, 147–151 (2004).

8. Schneider, L. *et al.* Variability of Labrador Sea Water transported through Flemish Pass during 1993–2013. *J. Geophys. Res. Oceans* **120**, 5514–5533 (2015).

9. Good, S. A., Martin, M. J. & Rayner, N. A. EN4: Quality controlled ocean temperature and salinity profiles and monthly objective analyses with uncertainty estimates. *J. Geophys. Res. Oceans* **118**, 6704–6716 (2013).

10. McDougall, T. J. & Barker, P. M. *Getting Started with TEOS-10 and the Gibbs Seawater (GSW) Oceanographic Toolbox*. SCOR/IAPSO WG, 127(532), 1-28 (2011).

11. BAS, M. J. L., MAITRE, R. W. L., STRECKEISEN, A. & ZANETTIN, B. A Chemical Classification of Volcanic Rocks Based on the Total Alkali-Silica Diagram. *J. Petrol.* **27**, 745–750 (1986).

12. Lane, C. S. *et al.* Was the 12.1ka Icelandic Vedde Ash one of a kind? *Quat. Sci. Rev.* **33**, 87–99 (2012).

13. Heaton, T. J. *et al.* Marine20—The Marine Radiocarbon Age Calibration Curve (0–55,000 cal BP). *Radiocarbon* **62**, 779–820 (2020).

14. Rasmussen, S. O. *et al.* A new Greenland ice core chronology for the last glacial termination. *J. Geophys. Res.* **111**, D06102 (2006).

15. McCave, I. N. & Andrews, J. T. Distinguishing current effects in sediments delivered to the ocean by ice. I. Principles, methods and examples. *Quat. Sci. Rev.* **212**, 92–107 (2019).

16. Boyle, E. A. Manganese carbonate overgrowths on foraminifera tests. *Geochim. Cosmochim. Acta* **47**, 1815–1819 (1983).

17. Barker, S., Greaves, M. & Elderfield, H. A study of cleaning procedures used for foraminiferal Mg/Ca paleothermometry. *Geochem. Geophys. Geosyst.* **4**, 8407 (2003).

18. Sessford, E. G. *et al.* High-Resolution Benthic Mg/Ca Temperature Record of the Intermediate Water in the Denmark Strait Across D-O Stadial-Interstadial Cycles. *Paleoceanogr. Paleoclimatol.* **33**, 1169–1185 (2018).

19. Keul, N. *et al.* Exploring foraminiferal Sr/Ca as a new carbonate system proxy. *Geochim. Cosmochim. Acta* **202**, 374–386 (2017).

20. Yu, J., Foster, G. L., Elderfield, H., Broecker, W. S. & Clark, E. An evaluation of benthic foraminiferal B/Ca and δ11B for deep ocean carbonate ion and pH reconstructions. *Earth Planet. Sci. Lett.* **293**, 114–120 (2010).

21. Carlson, A. E. *et al.* Subtropical Atlantic salinity variability and Atlantic meridional circulation during the last deglaciation. *Geology* **36**, 991–994 (2008).

22. *The GEBCO_2014 Grid, version 20150318* (GEBCO Compilation Group, 2015).
