# Supplementary material for: Co-ordinated shifts in deep-water formation and Gulf Stream migration during abrupt climate changes
Source: Nat Commun. 2026 Jun 11;17:4966. doi: 10.1038/s41467-026-73832-4 (PMC13260390; doi:10.1038/s41467-026-73832-4)
Supplement: Supplementary file 2 — Transparent Peer Review file [file 41467_2026_73832_MOESM2_ESM.pdf]

nature portfolio

Peer Review File

Co-ordinated Shifts in Deep-water Formation and Gulf Stream Migration During Abrupt Climate Changes

Corresponding Author: Mr Fangjingcheng Zhu

This file contains all reviewer reports in order by version, followed by all author rebuttals in order by version.

Version 0:

Reviewer comments:

Reviewer #1

(Remarks to the Author)
Review of “Co-ordinated Shifts in Deep-water Formation and Gulf Stream Migration During Abrupt Climate Changes” by Zhu et al. for Nature Communications

The authors present several new palaeoceanographic records from marine sediments covering the last deglaciation. They use a suite of proxies and various locations across the Northwest Atlantic, thus enabling them to reconstruct oceanographic changes. These records have the potential to improve our understanding of past changes in the strength and position of ocean currents in the Northwest Atlantic, and with it to inform us about potential future changes in response to AMOC weakening. They find evidence that the u-NADW strengthened, l-NADW weakened, and Gulf Stream moved northward during the Younger Dryas. Additionally, their results can determine the sequence and lags behind these changes. These findings are novel and of interest to the palaeoclimate community and beyond to any area working on the implications of future AMOC weakening.

The analysis is comprehensive and appropriate for the research problem, drawing on multiple lines of evidence using multiple separate proxies. The results are also supported by published records and a climate model output, making the conclusions particularly robust. Overall, the manuscript is well-written but could benefit from some light editing (please see my specific comments).

My biggest concern is as follows: while I am convinced by the results, the methods behind the chronology should be better explained. The designated chronology section places the methods in their climatic context very well, but it lacks methodological detail. Some questions I had while reading it include: (1) why was 44GGC age model seen as correct and mapped onto 09GGC rather than the other way around, especially given the latter has more dates; (2) how robust is the correlation that the final age model is developed upon; and (3) how much were the three records warped from their original age models to improve this correlation? The intra-core, inter-proxy timings and correlations with other records show the results are robust, but the chronological methods should be clarified, and their uncertainties/assumptions should be explicitly mentioned in the text. Further detail on these concerns and some suggestions can be found under my specific comments.

Additionally, because of the diverse readership of this journal a map of all the currents and locations mentioned in text should be the first figure. Moving Extended Data Fig. 1 to the main text would help, but there are still omissions such as where Flemish Pass is (Line 141). I suggest moving this figure to be the new Figure 1 and scanning the text for any additional terms/locations that could be either added to the map or explained in its caption.

King regards.

Specific Comments on the Manuscript:

Line 28 – “northwards” to “northward”

Line 40 – add a hyphen (48±14-year)

Line 45 – add “Introduction” subtitle for clarity

Line 47 – “nutrient” to “nutrients”

Figure 1 – it would be useful to add another subplot (perhaps between a and b or b and c) showing the temporal offset applied during warping. This could be shown as the offset applied vs the initial age model chronological with a second line being the 95% uncertainties of the initial age model. Then readers could quickly gauge the level of warping applied to maximise this correlation and whether it is statistically robust

Lines 99-100 – sentence awkwardly worded

Lines 105-106 – sentence awkwardly worded

Line 120 – add “~” before 13.2

Line 123 – no space before “%”

Lines 129-130 – I may have missed it, but why are there two mean SS records per core?

Figure 2 – readers will be interested to see the age uncertainties for each record to understand the likelihood these trends are coincident. Perhaps add a floating error bar showing the mean age uncertainty for each record over this interval (like what you do in Fig. 1d and e)

Lines 152-153 – adding error bars to Fig. 2 will help support the claim that this is within age uncertainties

Lines 171-177 – readers may find it helpful if you discuss the recent evidence of this mechanism occurring on a smaller scale during the Little Ice Age. Please see:
Forman, E.C.G., Baldini, J.U.L., Jamieson, R.A. et al. The Gulf Stream moved northward at the end of the Little Ice Age. Commun Earth Environ 6, 552 (2025).
Lund, D. C., Lynch-Stieglitz, J. & Curry, W. B. Gulf Stream density structure and transport during the past millennium. Nature 444, 601–604 (2006).
Rashid, H., Zhang, Z. W., Piper, D. J. W., Patro, R. & Xu, Y. P. Impact of Medieval Climate Anomaly and Little Ice Age on the Labrador current flow speed and the AMOC reconstructed by the sediment dynamics and biomarker proxies. Palaeogeogr. Palaeoclimatol. Palaeoecol. 620, 111558 (2023).

Lines 183-189 – this sentence is too long. Multiple sentences across the manuscript could be broken down to improve flow (other examples include Lines 223-230 and 446-450). Please go through the manuscript checking for unnecessarily long sentences

Line 218 – reserve the word “records” for cores to avoid confusion. Please check the manuscript for other instances

Line 232 – add “a” in “with weakened AMOC”

Line 237 – remove the comma after “Gulf Stream”

Figure 4 – it would be useful to plot the points for 4a and 4c as well to show how robust record, and thus mid-point, is. Also, like with my comment for Fig. 2, it could be useful to show the typical age uncertainty for a-c vs d-e to show the certainty behind the coincident trends

Figure 4 caption – mention that the line style in g and i indicate relative strength

Lines 395-397 – to avoid potential circularity arguments, clarify that the correlation you mean is pre-warping. It would also be good to report the correlation statistics both pre- and post-warping here and elsewhere to support your methods

Lines 487-488 – sentence awkwardly worded. Should there be an “and” instead of the comma after “clay removal” to separate it from the leching steps? Should “for” be “using” or “by”?

Line 550 – 1,000 realisations sounds low. Are the results stable if this is raised?

References – there are multiple issues here. I suggest going through and carefully checking them. Some cite preprints rather than final papers. Article/page numbers are missing. Journal names are not always abbreviated, and some are in capitals letters.

Extended Data Table 1 – if 44GGC, 09GGC, and HU87003-7PC have 9, 17 (15 workable), and 2 dates respectively, why is 09GGC not used for the age model instead of 44GGC?

Extended Data Fig. 1 – colour scale bar missing units

Extended Data Fig. 2a – x axis label overlaps with tick labels

Extended Data Fig. 3 – looking at the age models and their dates, it looks to me like a mid-point chronology between 44GGC and 09GGC would potentially satisfy more ties, especially in 3c. I understand that these 14C ties may be erroneous, but your decision to warp the age model beyond their uncertainties in 3c should be discussed. Additionally, like with my comment on Fig. 1, readers will be interested to see how much warping was applied relative to chronological uncertainties. Perhaps an applied offset line could be added to 3a when looking at the altering of HU87003-7PC's age model

Extended Data Fig. 5 – colours are too close making it hard to read

Reviewer #2

(Remarks to the Author)
This is a strong and interesting manuscript, and the core idea of explicitly differentiating upper and lower NADW to obtain a more dynamical view of AMOC strength changes and their mechanisms is both timely and valuable. The focus on how u-NADW and l-NADW respond differently to perturbations, and how this links to Gulf Stream migration, significantly advances process-level understanding beyond bulk AMOC metrics.
Overall view:
The manuscript presents high-resolution sediment records from the Northwest Atlantic margin to reconstruct coordinated changes in u-NADW, l-NADW, and Gulf Stream position during the last deglaciation, with an emphasis on the Younger Dryas. The explicit separation of u-NADW and l-NADW responses, and the proposed sequence of circulation changes leading atmospheric and cryospheric responses, is conceptually powerful and aligns well with modern observational work that treats upper and lower NADW as dynamically distinct layers. The writing is generally clear, the multiproxy strategy is compelling, and the topic is highly relevant for understanding both past abrupt climate events and future AMOC behavior.

My concerns and related question are listed below:
1. From local sortable silt to u-NADW strength: the logical step from "local near-bottom flow speed at ~1–2 km" to "basin-scale u-NADW strength" remains somewhat under-argued and could be made more robust.
a. The text currently assumes that, because the cores lie today within the u-NADW layer and beneath the Deep Western Boundary Current, changes in sortable silt necessarily reflect changes in u-NADW strength. It would help to more explicitly consider and, where possible, rule out alternative contributors to local flow variability, such as mesoscale recirculation, slope current variability, or changes in overlying Gulf Stream path and eddy activity that could alter shear and near-bottom stress without requiring a basin-wide change in u-NADW formation. Bruera et al. (2023) might provide some insight.
b. The authors could better explain why the chosen sites are expected to be particularly sensitive to changes in the strength of the u-NADW limb rather than more local circulation changes, perhaps by drawing on modern hydrographic/velocity sections or model output that show the dominance of the u-NADW layer at these depths and locations.
c. Given that the seesaw between u- and l-NADW is a central result, a short sensitivity discussion (or schematic) clarifying how much of the observed sortable silt signal would be expected from plausible changes in local circulation versus true u-NADW transport changes would strengthen the causal inference.
2. Interpreting local margin warming as Gulf Stream latitude shifts: the current argument of a northward shift of the Gulf Stream is plausible and supported by the similarity to modelled and observed SST "dipole" patterns under AMOC weakening. Nevertheless, the chain of reasoning from local thermal anomalies to meridional Gulf Stream migration could be more critically examined.
a. The manuscript leans heavily on spatial analogies to modern SST dipoles and model experiments, plus the present-day proximity of the sites to the Gulf Stream front. It would be valuable to discuss more explicitly whether changes in eddy heat fluxes, shelf-slope current structure, or along-slope advection could, in principle, produce similar local warming at these cores without a large-scale meridional shift of the Gulf Stream path. This once again is a rule out exercise.
The reason being, in Molodtsov et al. (2025), observed multidecadal changes in North Atlantic temperature and salinity can be largely explained by external forcings (greenhouse gases, aerosols, volcanic eruptions, etc.), and that CMIP6 models tend to underestimate the amplitude or structure of these forced signals. This means that what might look like internally driven AMOC variability or purely "ocean-led" changes here can, in fact, be strongly shaped by the time-varying external forcing, making causal inferences based solely on internal dynamics potentially misleading.
b. If available, additional independent indicators of frontal position (e.g., faunal assemblages that track front location, or comparison with cores straddling the modern Gulf Stream path) could be highlighted more strongly, or the limits of reconstructing exact Gulf Stream latitude from the present data could be acknowledged.
c. It would help to explicitly discuss how robust the inferred 480-year and ~800–900-year lags are to plausible end-member reservoir-age and tie-point choices. For instance, what range of lag remains if one considers conservative bounds on reservoir age variability or alternative IRD–Ca^{2+} alignments?

References:
Molodtsov, S., Marinov, I., Weijer, W. et al. North Atlantic temperature and salinity changes are driven by external forcing, underestimated by CMIP6 models. npj Clim Atmos Sci 8, 332 (2025). https://doi.org/10.1038/s41612-025-01210-w
Bruera, R., Curbelo, J., García-Sánchez, G., & Mancho, A. M. (2023). Mixing and geometry in the North Atlantic Meridional Overturning Circulation. Geophysical Research Letters, 50, e2022GL102244. https://doi.org/10.1029/2022GL102244

Version 1:

Reviewer comments:

Reviewer #1

(Remarks to the Author)
The authors have carefully addressed all my concerns from the first round of reviews. These changes have greatly improved the flow and clarity of the manuscript. I only have a few of typographical suggestions:
1. Line 89: “shown in black dots” should be “shown as black dots”
2. Fig. 5e: the “Sea ice expansion” arrow is slightly overlapping
3. Throughout the manuscript ranges should use en dashes instead of hyphens (e.g., Line 680 and 784)
Thank you for taking the time to thoroughly address all my comments.

Reviewer #2

(Remarks to the Author)
The authors have substantially improved the manuscript and, in my view, have satisfactorily addressed the major issues raised in the first round, both by myself and by the other reviewer. The revised paper is now much clearer in its treatment of chronology, the linkage between sortable silt and u NADW strength, and the interpretation of Gulf Stream migration and phasing.
In my view, the revised explanation for why the sortable silt records reflect changes in u NADW strength is now broadly convincing and adequate for this journal, but it still rests on several reasonable assumptions that should remain explicit. The authors have done a good job showing, with modern hydrography and velocity sections, that the cores sit in the present day u NADW density/velocity core rather than in slope water, and they carefully argue against alternative local controls such as slope water variability, recirculation changes, or enhanced eddy activity, supported by the coherence with independent regional proxies. At the same time, interpreting these boundary current records as indicative of basin scale u NADW and AMOC changes necessarily involves extrapolating from a limited set of sites and assuming that late deglacial water mass structure is sufficiently analogous to the modern. I am comfortable with these inferences, but I would encourage the authors to keep the wording slightly cautious (e.g. “we infer”, “our records are consistent with basin scale changes”) when referring to u NADW and AMOC at the basin scale, to signal to readers that the conclusions are built from strong regional evidence plus physically motivated, but not directly observed, assumptions.
My remaining comments are relatively minor and mostly relate to wording and scope:
• Similarly, the discussion of a “northward shift” of the Gulf Stream is now much better justified, but the data most robustly demonstrate an increased influence of Gulf Stream–derived waters and a dipole-like pattern, rather than a uniquely determined meridional path change. It may be helpful to emphasize that, with current core coverage, the results point to a northward displacement and/or strengthening of the Gulf Stream influence in this region, and that finer details of path geometry must await denser spatial sampling.
• The extension of the deglacial mechanism to industrial era and future AMOC changes is interesting and well signposted as a hypothesis. I would just recommend ensuring that this forward-looking discussion is clearly distinguished from the core, data driven deglacial results (e.g. a brief reminder that the industrial-era interpretation remains to be tested with robust l NADW reconstructions).
Recommendation
Overall, I think the authors have done an excellent job of addressing the previous round of comments. The chronology is now treated with appropriate methodological rigor and transparency, the physical interpretation of the sortable silt and temperature proxies is much more tightly argued, and the causal narrative is expressed with a level of caution that matches the strength of the evidence. I am happy with the revisions and support publication after the authors make any minor wording adjustments they and the editor deem necessary.

Open Access This Peer Review File is licensed under a Creative Commons Attribution 4.0 International License, which permits use, sharing, adaptation, distribution and reproduction in any medium or format, as long as you give appropriate credit to the original author(s) and the source, provide a link to the Creative Commons license, and indicate if changes were made.
In cases where reviewers are anonymous, credit should be given to 'Anonymous Referee' and the source.
The images or other third party material in this Peer Review File are included in the article's Creative Commons license, unless indicated otherwise in a credit line to the material. If material is not included in the article's Creative Commons license and your intended use is not permitted by statutory regulation or exceeds the permitted use, you will need to obtain permission directly from the copyright holder.
To view a copy of this license, visit https://creativecommons.org/licenses/by/4.0/

Response to referees: "Co-ordinated Shifts in Deep-water Formation and Gulf Stream Migration During Abrupt Climate Changes" by Fangjingcheng Zhu, Alice Carter-Champion, Jack H. Wharton, Joel Bracamontes-Ramírez, Andrea Burke, Peter B. de Menocal, David Fairman, Lloyd D. Keigwin, Thomas M. Marchitto, Eirini Papachristopoulou, James W. B. Rae, Yair Rosenthal, Ning Zhao, and David J. R. Thornalley.

Author's note – our detailed responses are given below in blue, with relevant direct quotes from the manuscript included where appropriate (*blue italics*). We have also included updated figures where appropriate.

Reviewer #1 (Remarks to the Author):

Review of "Co-ordinated Shifts in Deep-water Formation and Gulf Stream Migration During Abrupt Climate Changes" by Zhu et al. for Nature Communications

The authors present several new palaeoceanographic records from marine sediments covering the last deglaciation. They use a suite of proxies and various locations across the Northwest Atlantic, thus enabling them to reconstruct oceanographic changes. These records have the potential to improve our understanding of past changes in the strength and position of ocean currents in the Northwest Atlantic, and with it to inform us about potential future changes in response to AMOC weakening. They find evidence that the u-NADW strengthened, l-NADW weakened, and Gulf Stream moved northward during the Younger Dryas. Additionally, their results can determine the sequence and lags behind these changes. These findings are novel and of interest to the palaeoclimate community and beyond to any area working on the implications of future AMOC weakening.

The analysis is comprehensive and appropriate for the research problem, drawing on multiple lines of evidence using multiple separate proxies. The results are also supported by published records and a climate model output, making the conclusions particularly robust. Overall, the manuscript is well-written but could benefit from some light editing (please see my specific comments).

We thank the reviewer for their supportive comments and have addressed their suggestions, as detailed below.

My biggest concern is as follows: while I am convinced by the results, the methods behind the chronology should be better explained. The designated chronology section places the methods in their climatic context very well, but it lacks methodological detail.

We thank the reviewer for this helpful comment and agree that the paper will benefit from clearer explanation of methodological details. We have therefore revised and expanded the relevant section of the Methods to clarify these aspects, including explicitly outlining the assumptions underlying the age models (see detailed responses below) and detailing how chronological uncertainties are propagated using Bayesian *P_Sequence* depositional models implemented in OxCal v4.4 (Methods). For

clarity, we have also renamed the two age models in our study (initial and final age models) to Radiocarbon-based and Tuned age models, respectively. The revised text is reproduced below:

"Radiocarbon-based age models were thus constructed using Bayesian P_Sequence depositional models produced in OxCal v4.4[90] with a variable k factor and individual Delta_R functions set for each date, to allow for time-varying offsets in sedimentation rate. A 'general' outlier model (5%) was also specified for each depth except for records with the Vedde Ash tephra in, as there is a well-established independent age for this tephra[26] (Supplementary Fig. 3)."

"Uncertainties of the Tuned age models were estimated using the P_Sequence depositional models: uncertainties within the tie-points to the NGRIP record were estimated visually and chronological uncertainty errors were propagated with the overall uncertainty on the NGRIP records[78]. These were then input as a C_Date for the deeper portion of the record and supplemented with radiocarbon dates from the Holocene, where available, with a modern ΔR calculated from the 'deltar' calculator[95] (see Supplementary Fig. 3)."

Additionally, we thank the reviewer for the opportunity to review our methods. In doing so, we identified one inaccurate assumption in original manuscript: the use of modern regional reservoir ages (ΔR) to correct the radiocarbon dates in our Radiocarbon-based age models. This assumption is inappropriate considering the temporal and spatial variability of ΔR during the deglaciation (Skinner et al., 2019). We have therefore revised the Radiocarbon-based age modelling approach and instead constrain ΔR in the Radiocarbon-based age models using the reservoirs age estimated from the Vedde Ash tephra layer present in both cores. The revised Methods text is reproduced below:

"^{14}C dates were calibrated using the Marine20 calibration curve[33] to account for variations in both atmospheric ^{14}C concentration and global marine reservoir age, corrected with regional reservoir ages (ΔR) estimated from Vedde Ash tephra layer with uncertainties to account for variable ΔR throughout the deglaciation."

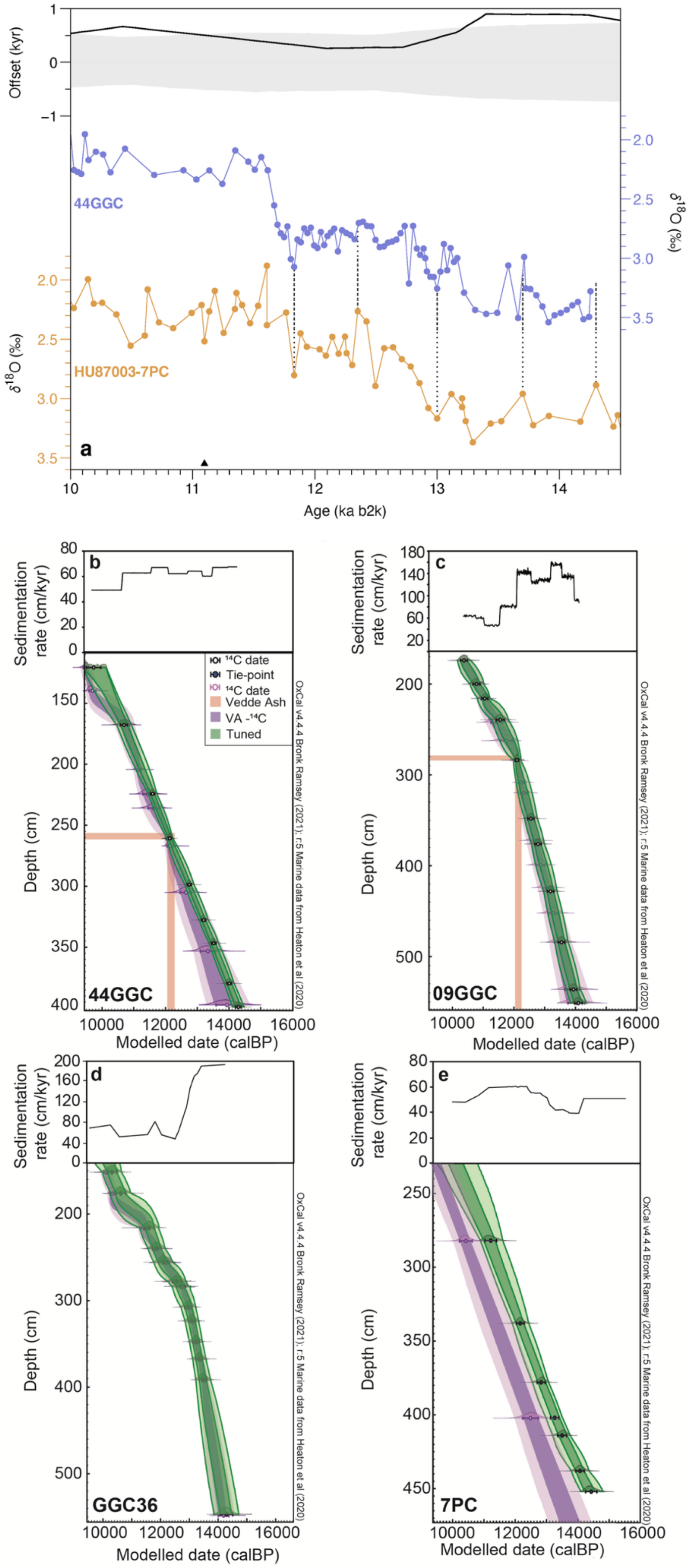

Offset (kyr)
44GGC
HU87003-7PC
δ18O (‰)
Age (ka b2k)
a
b
c
d
e
Sedimentation rate (cm/kyr)
Depth (cm)
Modelled date (calBP)
14C date
Tie-point
Vedde Ash
VA -14C
Tuned
44GGC
09GGC
GGC36
7PC
OxCal v4.4.4 Bronk Ramsey (2021); r:5 Marine data from Heaton et al (2020)

***Supplementary Fig 3. Chronology and age-depth relationship. a** Upper: Temporal offset between the Radiocarbon-based and Tuned age models of HU87003-7PC (black line). Also shown is the uncertainty range of the Radiocarbon-based age model (grey shading; 95% confidence interval, CI). Lower: Np $\delta^{18}O$ of 44GGC (blue) and HU87003-7PC (yellow). The age model of HU87003-7PC was based on correlation of Np $\delta^{18}O$ with 44GGC (dotted lines) and a ^{14}C date calibrated using Marine20[13] (black triangle). **b** Upper: Sedimentation rate of 44GGC. Lower: Depth vs age of 44GGC. Dark purple and dark green shadings denote 68% confidence intervals of the Radiocarbon-based and Tuned age models, respectively. Pale purple and pale green shadings denote 95% confidence intervals of the Radiocarbon-based and Tuned age models, respectively. Calibrated ^{14}C dates used in the Radiocarbon-based age model are shown in purple circles with error bars denoting ±1σ error. Stratigraphic tie points (filled black circles) and calibrated ^{14}C dates (open black circles) used in the Tuned age model are shown with error bars denoting ±1σ error. The pale red shadings denote the Vedde Ash tephra layer with ±2σ error[14]. **c-e** As in **(b)** but for 09GGC, GGC36, and HU87003-7PC, respectively.*

Some questions I had while reading it include: (1) why was 44GGC age model seen as correct and mapped onto 09GGC rather than the other way around, especially given the latter has more dates;

We thank the reviewer for this thoughtful question and agree that the assumptions underlying the age model construction should be explained more explicitly. We selected 44GGC (rather than 09GGC) as the primary target for establishing the age model for two main reasons: (1) 44GGC was the primary focus of the study because its location is ideally suited for tracking Gulf Stream migration (Fig. 1 shows that it is situated near the boundary between the cold Labrador Current and warm Gulf Stream); (2) the more southwesterly position of 44GGC results in a clearer 'on-off' behaviour in ice-rafted detritus deposition between cold and warm periods. In contrast, 09GGC records more frequent ice-rafting events and therefore exhibits a more saturated signal (Supplementary Fig. 4). Furthermore, the clearer IRD signal in 44GGC provides the basis for correlation with the NGRIP chronology. To clarify why the age model for 44GC was transferred to 09GGC, we have added the following text to the Methods and additional supplementary figure:

"Notably, 44GGC (rather than 09GGC) was selected as the primary target for establishing the Tuned age model for two main reasons: (1) 44GGC is situated ideally for tracking Gulf Stream migration (Fig. 1a); (2) The more south-westerly position of 44GGC enables it to document a cleaner 'on-off' signal for IRD deposition between cold and warm periods (Supplementary Fig. 4a, b) than compared to core 09GGC which is affected by more frequent drifting icebergs/sea ice and therefore exhibits lower signal-to-noise ratios (Supplementary Fig. 4d, e). Furthermore, the clearer IRD signal in 44GGC provides the basis for correlation with the NGRIP chronology (Fig. 2c, d)."

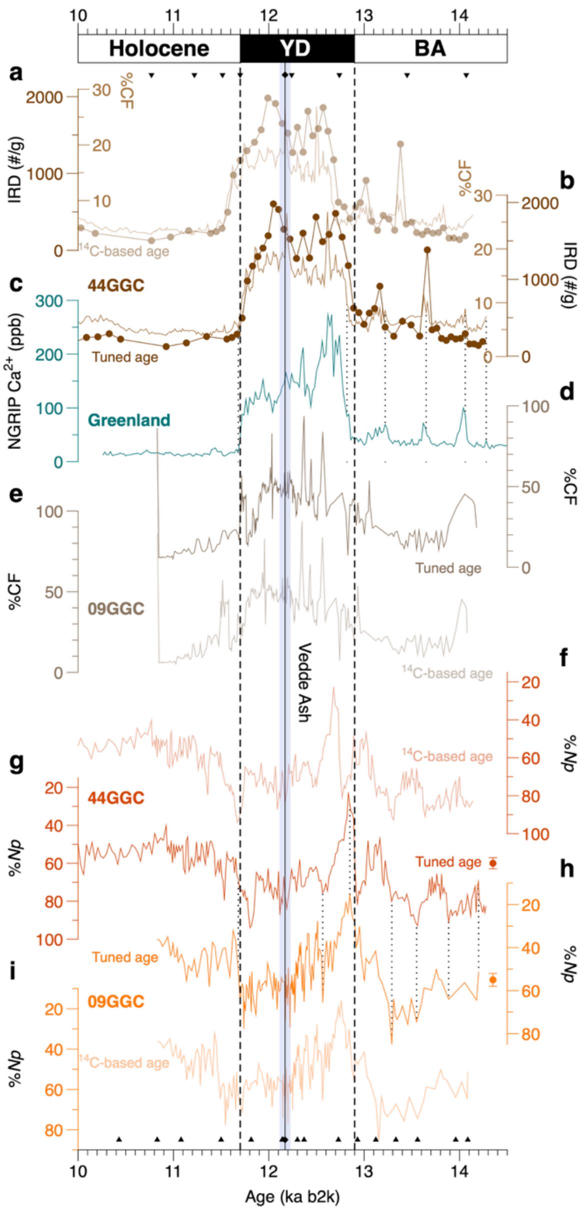

Supplementary Fig 4. Radiocarbon-based and Tuned age models of 44GGC and 09GGC. a *44GGC %CF (light brown line) and IRD (dark brown line with dots) plotted on the Radiocarbon-based age model based on a tephra tie point (diamond) and ^{14}C dates (inverted triangles) calibrated using the Marine20 calibration curve[13], corrected with regional reservoir age (ΔR) estimated from Vedde Ash tephra layer with uncertainties to account for variable ΔR throughout the deglaciation.* ***b*** *Same as in (**a**) but plotted on the Tuned age model using the tephra tie point (vertical solid line) and the correlation of %CF and IRD with NGRIP Ca^{2+} (tie points indicated by dotted lines).* ***c*** *NGRIP Ca^{2+}[14].* ***d*** *09GGC %CF plotted on the Tuned age model.* ***e*** *09GGC %CF plotted on the Radiocarbon-based age model.* ***f*** *44GGC %Np plotted on the Radiocarbon-based age model.* ***g*** *44GGC %Np plotted on the Tuned age model.* ***h*** *09GGC %Np plotted on the Tuned age model synchronized with 44GGC using the tephra tie point (vertical solid line) and correlation with 44GGC %Np (tie points indicated by dotted lines). The error bars in **g** and **h** denote averaged ±1σ errors.* ***i*** *09GGC %Np plotted on the Radiocarbon-based age model based on a tephra tie point (diamond) and ^{14}C dates (triangles) calibrated using the Marine20 calibration curve[13], corrected with ΔR estimated from Vedde Ash tephra layer*

with uncertainties to account for variable ΔR throughout the deglaciation. The age of the Vedde Ash tephra layer is indicated in a solid vertical line with shading denoting ±1σ error[14].

(2) how robust is the correlation that the final age model is developed upon;

We agree that it is important to statistically demonstrate the robustness of the Tuned age model by quantifying the correlation. We have therefore quantified the correlation between the NGRIP Ca^{2+} and 44GGC %CF for both the Radiocarbon-based and Tuned age models. We then propagated age uncertainties using 10,000 Monte Carlo age perturbations and recalculated the correlation after randomly perturbing ages within ±1σ error. Taken together, the correlation results show a statistically significant improvement in the Tuned age model. We have therefore added the following text in the Methods and added an additional supplementary figure to demonstrate the robustness of the Tuned age model:

"The robustness of the Tuned age model is further supported by the correlation between the NGRIP Ca^{2+} and 44GGC %CF (Fig. 2c, d; Supplementary Fig. 12). We compared the correlation between 44GGC %CF and NGRIP Ca^{2+} under the Radiocarbon-based and Tuned age models by interpolating both records onto a 20-year time step, which is plausible with respect to the resolution of both records. As expected, the correlation increases from r = 0.69 to r = 0.90 under the Tuned age model because the latter is aligned to the NGRIP record (Supplementary Fig. 12a-d). A Fisher r-to-z test confirms that this improvement is statistically significant; this was calculated using a coarser temporal resolution (100-year time step) to minimise sensitivity to the interpolation timestep (z = 2.06, p = 0.04). We then propagated chronological uncertainty using 10,000 Monte Carlo age perturbations, recalculating the correlation after randomly perturbing ages within their 1σ uncertainties (Supplementary Fig. 12e, f). Under the Radiocarbon-based age model the correlation varies widely (median r = 0.66, 95% range 0.49-0.76), indicating that the inferred alignment is sensitive to associated age uncertainties. In contrast, the Tuned age model yields a consistently strong relationship (median r = 0.86, 95% range 0.81-0.89), and in all realizations the correlation exceeds that obtained under the Radiocarbon-based chronology. Taken together, this suggests that the Tuned age model is robust."

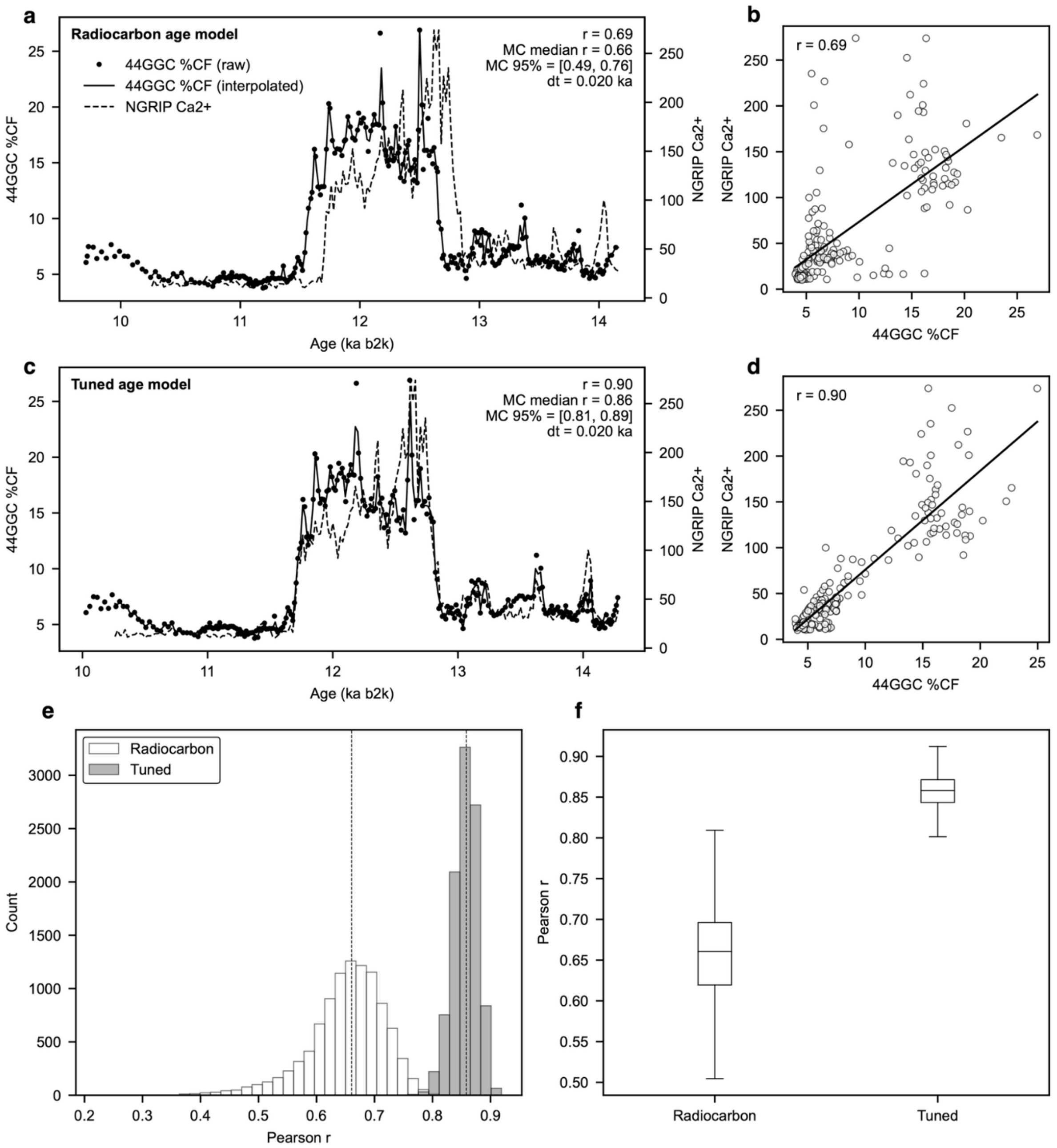

Supplementary Fig 12. Correlation analysis for NGRIP Ca^{2+} vs 44GGC %CF under the Radiocarbon-based and Tuned age models. a *Time series of NGRIP Ca^{2+} (dashed line)*[14] *and 44GGC %CF under the Radiocarbon-based age model (raw data: dots; interpolated data: solid line).* ***b*** *Scatter plot for interpolated NGRIP Ca^{2+} and 44GGC %CF under the Radiocarbon-based age model.* ***c*** *As in* ***a*** *but for the Tuned age model.* ***d*** *As in* ***b*** *but for the Tuned age model.* ***e*** *Monte Carlo distributions of correlation coefficients (r) between NGRIP Ca^{2+} and 44GGC %CF under the Radiocarbon-based (white) and Tuned (grey) age models, obtained by randomly perturbing ages within 1σ uncertainties.* ***f*** *Box-and-whisker representation of the same Monte Carlo correlation distributions shown in* ***e****, for the Radiocarbon-based (left) and Tuned (right) age models.*

and (3) how much were the three records warped from their original age models to improve this correlation?

We agree that it is useful to visualize the differences between the Radiocarbon-based and Tuned age models and have therefore included the age uncertainties in the revised Supplementary Fig. 3 (see above). We have also followed the reviewer's suggestion to add one additional panel (b) in Fig. 2, which illustrates the degree of warping applied to the Tuned age model of 44GGC (below). Both plots indicate that the 'warping' applied to the Tuned age models fall within the uncertainty ranges of the Radiocarbon-based age models.

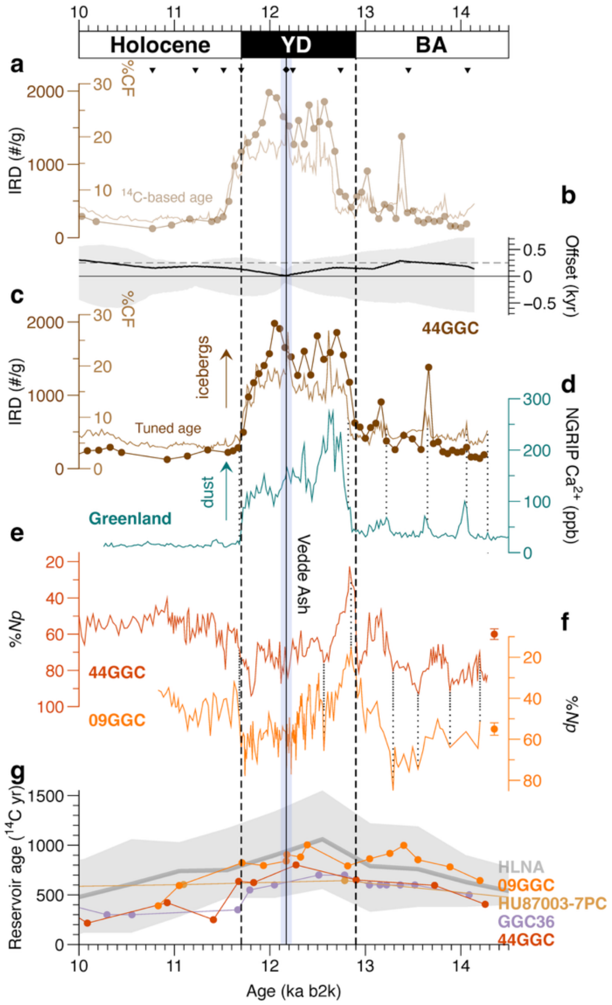

***Fig 2. Radiocarbon-based and Tuned age models – linked to Greenland ice core chronology. a** 44GGC %CF (light brown line) and IRD (dark brown line with dots) plotted on the Radiocarbon-based age model based on a tephra tie point (diamond) and ^{14}C dates (inverted triangles) calibrated using the Marine20 calibration curve[33], corrected with regional reservoir age (ΔR) estimated from Vedde Ash tephra layer with uncertainties to account for variable ΔR throughout the deglaciation. **b** Temporal offset between the Radiocarbon-based and Tuned age models of 44GGC (black line). Also shown is the uncertainty range of the Radiocarbon-based age model (grey shading; 95% confidence interval, CI). **c** Same as in **a** but plotted on the Tuned age model using the tephra tie point (vertical solid line) and the correlation of %CF and IRD with NGRIP Ca^{2+} (tie points indicated by dotted lines). **d** NGRIP Ca^{2+}[26]. **e** 44GGC %Np plotted on the Tuned age model. **f** 09GGC %Np plotted on the Tuned age model synchronized with 44GGC using the tephra tie point (vertical solid line) and correlation with 44GGC %Np (tie points indicated by*

*dotted lines). The error bars in **e** and **f** denote averaged ±1σ errors. **g** Surface ^{14}C reservoir ages of 44GGC (red), 09GGC (orange) and HU87003-7PC (gold) calculated using the stratigraphic age constraints. Note the chronology of HU87003-7PC was synchronized with 44GGC by correlating Np $\delta^{18}O$ (Supplementary Fig. 3a). Also shown is the regionally averaged high-latitude North Atlantic (HLNA) surface ^{14}C reservoir age[32] (thick grey line) with 95% CI (light grey shading). The regional surface reservoir ages were then applied to GGC36 (purple) and used for correcting its ^{14}C dates[34] (Supplementary Fig. 3d). The age of the Vedde Ash tephra layer is indicated in a solid vertical line with shading denoting ±1σ error[26].*

The intra-core, inter-proxy timings and correlations with other records show the results are robust, but the chronological methods should be clarified, and their uncertainties/assumptions should be explicitly mentioned in the text. Further detail on these concerns and some suggestions can be found under my specific comments.

We thank the reviewer for these specific comments, which are addressed in the final section of this response.

Additionally, because of the diverse readership of this journal a map of all the currents and locations mentioned in text should be the first figure. Moving Extended Data Fig. 1 to the main text would help, but there are still omissions such as where Flemish Pass is (Line 141). I suggest moving this figure to be the new Figure 1 and scanning the text for any additional terms/locations that could be either added to the map or explained in its caption.

We agree with the reviewer that including a map improves the clarity of the manuscript. Accordingly, we have moved Extended Data Fig. 1 to Fig. 1 in the main text. We have also added a new panel (b) showing a bathymetric map, with major topographic features and other elements labelled as suggested by the reviewer (see below). Additional hydrographic panels have also been added in response to Reviewer 2's comments.

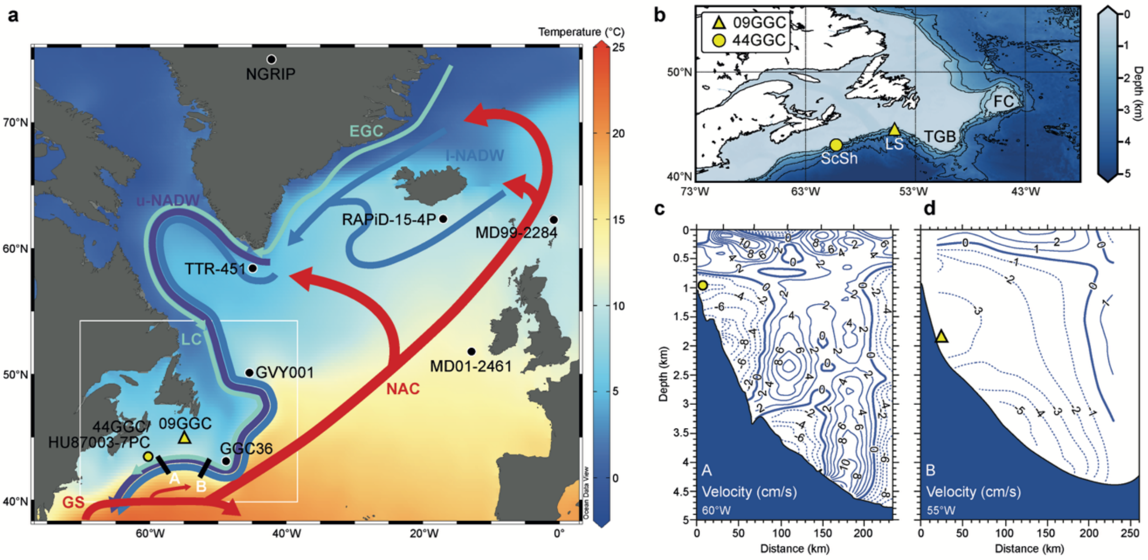

Fig 1. Locations of study sites and modern ocean conditions in the North Atlantic. a *Map shows annual mean sea surface temperature[19] (colour shading) and surface and deep circulations[20] (arrows) in the North Atlantic. Locations of 44GGC (966 m) and HU87003-7PC (920 m) are shown in yellow dots. Location of 09GGC (1854 m) is shown in yellow triangle. Other study sites discussed in this paper are shown in black dots: GGC36 (1520 m), GVY001 (3721 m), MD01-2461 (1153 m), TTR-451 (1927 m), RAPiD-15-4P (2133 m), MD99-2284 (1500 m), and North Greenland Ice Core Project (NGRIP). Black lines denote locations of the cross sections at 60°W (A) and 55°W (B) shown in panels* ***c*** *&* ***d****, respectively. GS Gulf Stream, NAC North Atlantic Current, EGC East Greenland Current, LC Labrador Current, u/l-NADW upper/lower-North Atlantic Deep Water. The map was generated using Ocean Data View[21].* ***b*** *Bathymetry map of the Northwest Atlantic, where locations of 44GGC and 09GGC are shown in yellow circle and triangle, respectively. FC Flemish Cap, TGB Tail of Grand Banks, LS Laurentian Slope, ScSh Scotian Shelf.* ***c*** *Absolute geostrophic velocity at 60°W[22] (section A in* ***a****). Location of 44GGC is marked in yellow circle.* ***d*** *Absolute geostrophic velocity at 55°W[23,24] (section B in* ***a****). Location of 09GGC is marked in yellow triangle. In* ***c*** *and* ***d****, positive values indicate poleward flow. Note both 44GGC and 09GGC are located in the core of the u-NADW flow.*

Specific Comments on the Manuscript:

Line 28 – “northwards” to “northward” Corrected.

Line 40 – add a hyphen (48±14-year) Added.

Line 45 – add “Introduction” subtitle for clarity Added.

Line 47 – “nutrient” to “nutrients” Corrected.

Figure 1 – it would be useful to add another subplot (perhaps between a and b or b and c) showing the temporal offset applied during warping. This could be shown as the offset applied vs the initial age model chronological with a second line being the 95% uncertainties of the initial age model. Then readers could quickly gauge the level of warping applied to maximise this correlation and whether it is statistically robust Added.

Lines 99-100 – sentence awkwardly worded Corrected.

Lines 105-106 – sentence awkwardly worded Corrected.

Line 120 – add “~” before 13.2 Added.

Line 123 – no space before “%” Corrected.

Lines 129-130 – I may have missed it, but why are there two mean SS records per core?

For thoroughness, the grain size was run on two different machines (Coulter Counter and Malvern Mastersizer), of which the data show good agreement. In Fig. 3a, b, the lines with

solid circles are mean SS data generated using the Coulter Counter and the lines with open circles represent data generated using the Malvern Mastersizer.

Figure 2 – readers will be interested to see the age uncertainties for each record to understand the likelihood these trends are coincident. Perhaps add a floating error bar showing the mean age uncertainty for each record over this interval (like what you do in Fig. 1d and e)
Lines 152-153 – adding error bars to Fig. 2 will help support the claim that this is within age uncertainties

We agree that including error bars showing the mean age uncertainties improves the clarity of the figure. We have therefore added floating error bars indicating the mean age uncertainty (±1 σ) for each record (below). We have also added vertical error bars to panel a and b to show the average analytical error (±1 σ) of the SS data.

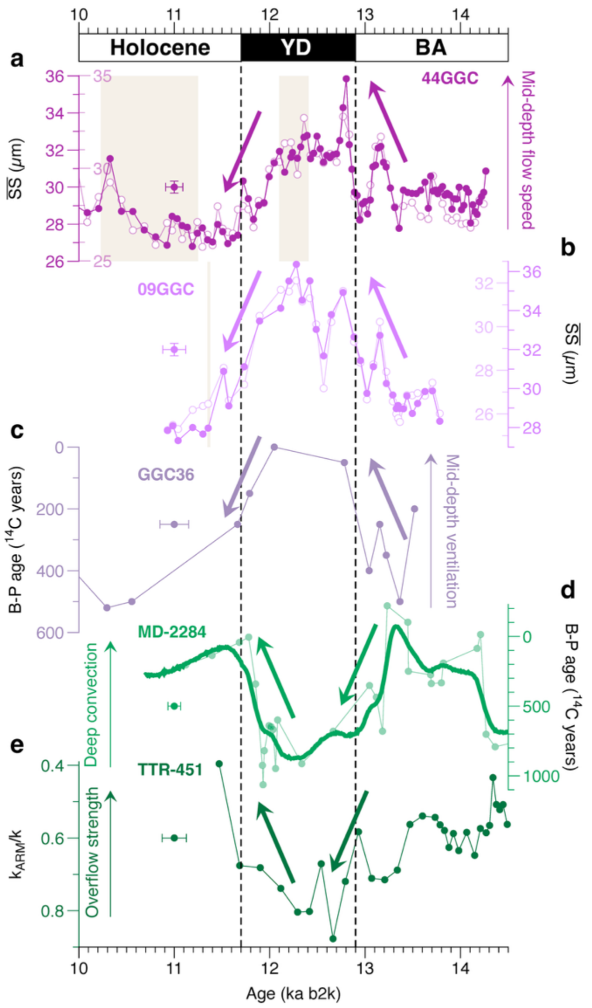

Fig 3. Seesaw relationship between the u-NADW and l-NADW during the last deglaciation. a *44GGC* $\overline{SS}$ *reflecting mid-depth flow speed.* ***b*** *09GGC* $\overline{SS}$ *reflecting mid-depth flow speed.* $\overline{SS}$ *data were measured on Coulter (line with solid dots) and Malvern (line with open dots). Light beige shadings denote intervals where* $\overline{SS}$ *data do not pass the validity test[39] (Supplementary Fig. 5), indicating poor sorting and unreliable records.* ***c*** *GGC36 B-P ^{14}C age[34] reflecting mid-depth ventilation.* ***d*** *MD99-2284 B-P ^{14}C age[40]*

reflecting Nordic Seas deep convection. ***e*** *TTR-451 κ_{ARM}/κ[41] reflecting strength of Nordic Seas overflow (l-NADW). All published records are reported in their original age models except* ***c*** *GGC36, where the surface ^{14}C reservoir age from the present study was applied to the ^{14}C dates to account for regional reservoir effects (Fig. 2g; Supplementary Fig. 3d). In* ***a-e****, horizontal floating error bars denote average age uncertainties (±1σ). In* ***a, b****, vertical floating error bars denote average analytical error (±1σ).*

Lines 171-177 – readers may find it helpful if you discuss the recent evidence of this mechanism occurring on a smaller scale during the Little Ice Age. Please see:
Forman, E.C.G., Baldini, J.U.L., Jamieson, R.A. et al. The Gulf Stream moved northward at the end of the Little Ice Age. Commun Earth Environ 6, 552 (2025).
Lund, D. C., Lynch-Stieglitz, J. & Curry, W. B. Gulf Stream density structure and transport during the past millennium. Nature 444, 601–604 (2006).
Rashid, H., Zhang, Z. W., Piper, D. J. W., Patro, R. & Xu, Y. P. Impact of Medieval Climate Anomaly and Little Ice Age on the Labrador current flow speed and the AMOC reconstructed by the sediment dynamics and biomarker proxies. Palaeogeogr. Palaeoclimatol. Palaeoecol. 620, 111558 (2023).

We agree that it would be helpful to refer to recent evidence for smaller-scale variability such as during the Little Ice Age. We have therefore added the following sentence to the manuscript:

"Paleoceanographic evidence from this region[11,57–60] has often invoked this mechanism for recent centennial climate variability."

Lines 183-189 – this sentence is too long. Multiple sentences across the manuscript could be broken down to improve flow (other examples include Lines 223-230 and 446-450). Please go through the manuscript checking for unnecessarily long sentences

We agree with the reviewer that several sentences throughout the manuscript could be shortened to improve clarity. We have therefore reviewed the entire manuscript and revised unnecessarily long sentences.

Line 218 – reserve the word "records" for cores to avoid confusion. Please check the manuscript for other instances Corrected.

Line 232 – add "a" in "with weakened AMOC" Added.

Line 237 – remove the comma after "Gulf Stream" Removed.

Figure 4 – it would be useful to plot the points for 4a and 4c as well to show how robust record, and thus mid-point, is. Also, like with my comment for Fig. 2, it could be useful to show the typical age uncertainty for a-c vs d-e to show the certainty behind the coincident trends

We agree with the reviewer that plotting the individual data points and showing the typical age uncertainties for each record strengthens the robustness of our interpretations. We have therefore revised Fig. 5 accordingly (see below):

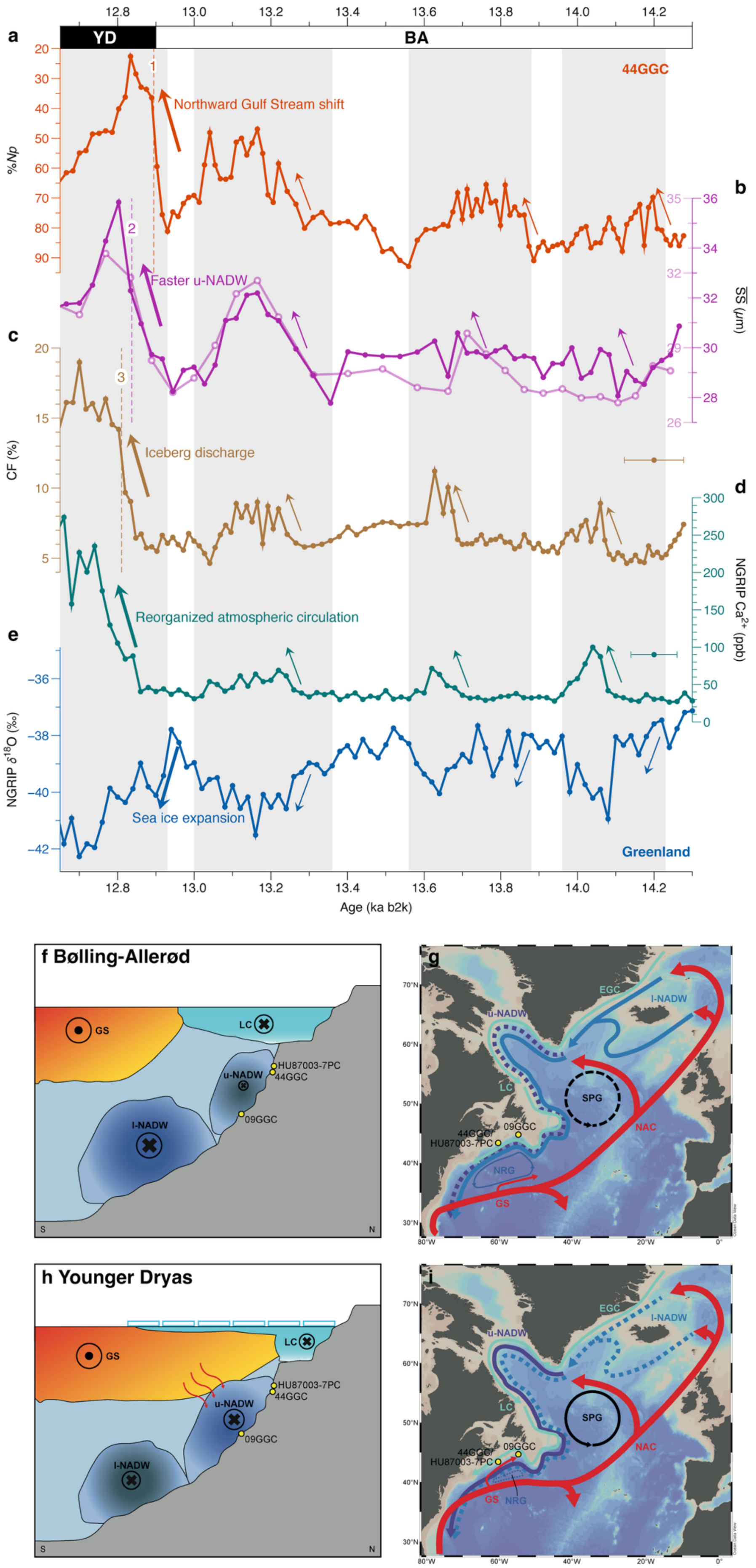
a
YD
BA
44GGC
%Np
Northward Gulf Stream shift
Faster u-NADW
b
SS (μm)
c
CF (%)
Iceberg discharge
d
NGRIP Ca2+ (ppb)
Reorganized atmospheric circulation
e
NGRIP δ18O (‰)
Sea ice expansion
Greenland
Age (ka b2k)
f Bølling-Allerød
GS
LC
u-NADW
l-NADW
HU87003-7PC
44GGC
09GGC
S
N
g
EGC
l-NADW
u-NADW
LC
SPG
NAC
NRG
GS
44GGC
09GGC
HU87003-7PC
h Younger Dryas
GS
LC
u-NADW
l-NADW
HU87003-7PC
44GGC
09GGC
S
N
i
EGC
l-NADW
u-NADW
LC
SPG
NAC
GS
NRG
44GGC
09GGC
HU87003-7PC

Fig 5. Co-ordinated ocean-atmospheric-ice response to abrupt deglacial climate change. a-c *Downcore proxy records of 44GGC.* ***a*** *%Np.* ***b*** $\overline{SS}$ *measured on Coulter (line with solid dots) and Malvern (line with open dots).* ***c*** *Percentage of coarse fraction (a proxy for IRD; Methods). Vertical dashed lines in* ***a-c*** *indicate the mean age of the mid-point for BA-YD transition for each proxy derived from bootstrapping analysis (Methods).* ***d*** *NGRIP* Ca^{2+26}*.* ***e*** *NGRIP* $\delta^{18}O^{26}$*. Vertical grey shadings denote centennial-scale Greenland cold events as shown in* ***Fig. 4****. In* ***c,d****, horizontal floating error bars denote average absolute age uncertainties (±1σ).* ***f-i*** *Schematic diagrams showing inferred changes in North Atlantic circulation during abrupt deglacial climate change.* ***f*** *The cross-section of the Northwest Atlantic shelf region during BA (Greenland warm intervals), where the surface ocean is likely dominated by cold, fresh Labrador Current and subsurface is occupied by strong l-NADW and relatively weaker u-NADW.* ***g*** *Inferred North Atlantic circulation during BA (Greenland warm intervals), with strong northern recirculation gyre, southernly positioned Gulf Stream, strong l-NADW, weak SPG and u-NADW.* ***h*** *As in (****f****) but for the scenario of YD, with a northward shifted Gulf Stream that subducts underneath a freshwater cap in the surface. In the subsurface, the l-NADW and u-NADW are likely weakened and strengthened, respectively, with warm Gulf Stream-derived water entrained in the mid-depths.* ***i*** *As in (****g****) but for YD, with weakened northern recirculation gyre, northward shifted Gulf Stream, weakened l-NADW, strengthened SPG and u-NADW. GS Gulf Stream, LC Labrador Current, u/l-NADW upper/lower-North Atlantic Deep Water, NRG northern recirculation gyre, NAC North Atlantic current, SPG subpolar gyre, EGC East Greenland current. The line styles in* ***g*** *and* ***i*** *indicate relative strength, with solid and dashed/dotted lines reflecting stronger and weaker strengths, respectively.*

Figure 4 caption – mention that the line style in g and i indicate relative strength Added.

Lines 395-397 – to avoid potential circularity arguments, clarify that the correlation you mean is pre-warping. It would also be good to report the correlation statistics both pre- and post-warping here and elsewhere to support your methods

We thank the reviewer for the constructive suggestions, and we agree it is useful to report the correlation statistics both pre- and post-warping to support our methods. We have therefore added detailed statistical analysis to evaluate the robustness of our Tuned age model by quantifying the correlation between NGRIP Ca^{2+} and 44GGC %CF under both the Radiocarbon-based and Tuned age models, followed by recalculating the correlation after randomly perturbing ages within ±1σ uncertainties using 10,000 Monte Carlo realizations. Our results suggest that our Tuned age model is statistically robust (see detailed responses above).

Lines 487-488 – sentence awkwardly worded. Should there be an "and" instead of the comma after "clay removal" to separate it from the leching steps? Should "for" be "using" or "by"? Corrected.

Line 550 – 1,000 realisations sounds low. Are the results stable if this is raised?

We agree that the robustness of the mid-point analysis benefits from a larger number of realizations. We have therefore repeated the analysis using 10,000 bootstrap realizations

while accounting for age model uncertainties (see below the response to Reviewer #2's comment 2c for more details). The lead-lag results remain stable when increasing 1,000 realizations (%*Np*-SS: 58±39 years; %*Np*-%CF: 84±52 years) to 10,000 realizations (%*Np*-SS: 58±38 years; %*Np*-%CF: 84±51 years) (95% CI).

References – there are multiple issues here. I suggest going through and carefully checking them. Some cite preprints rather than final papers. Article/page numbers are missing. Journal names are not always abbreviated, and some are in capitals letters.

We thank the reviewer for this helpful suggestion and agree that the reference list required more careful checking. We have now thoroughly reviewed and corrected the reference list.

Extended Data Table 1 – if 44GGC, 09GGC, and HU87003-7PC have 9, 17 (15 workable), and 2 dates respectively, why is 09GGC not used for the age model instead of 44GGC?

We thank the reviewer for the thoughtful question and have therefore responded in an earlier response above.

Extended Data Fig. 1 – colour scale bar missing units Corrected.

Extended Data Fig. 2a – x axis label overlaps with tick labels Corrected.

Extended Data Fig. 3 – looking at the age models and their dates, it looks to me like a mid-point chronology between 44GGC and 09GGC would potentially satisfy more ties, especially in 3c. I understand that these 14C ties may be erroneous, but your decision to warp the age model beyond their uncertainties in 3c should be discussed. Additionally, like with my comment on Fig. 1, readers will be interested to see how much warping was applied relative to chronological uncertainties. Perhaps an applied offset line could be added to 3a when looking at the altering of HU87003-7PC's age model

We thank the reviewer for their constructive suggestions. As we have already discussed in the responses above, we have now explicitly clarified the assumptions and uncertainties of our age models. The revised Supplementary Fig. 3 (above) also shows that the Tuned age models of all four cores are within the uncertainty ranges of their respective Radiocarbon-based age models. The reason that the previous Tuned age model of 09GGC appeared to be warped beyond its uncertainties in 3c was due to the inaccurate assumption in our original method, where we used modern ΔR and their uncertainties to correct the radiocarbon dates in our Radiocarbon-based age models. We have now revised the Radiocarbon-based age models by applying a ΔR estimated from the Vedde Ash tephra layer with uncertainties to account for variable ΔR throughout the deglaciation. Additionally, we have also followed the reviewer's suggestion by adding an applied offset line to indicate that the warping applied to HU87003-7PC was largely within the chronological uncertainties (see Supplementary Fig. 3a above). Note that the slightly larger offset in the uppermost and lowermost parts of the core was likely associated with the large uncertainties of the Radiocarbon-based age model, which was constrained by only two ^{14}C dates within this interval.

Extended Data Fig. 5 – colours are too close making it hard to read Corrected.

References
Skinner, L.C., Muschitiello, F. and Scrivner, A.E., 2019. Marine reservoir age variability over the last deglaciation: implications for marine carboncycling and prospects for regional radiocarbon calibrations. *Paleoceanography and Paleoclimatology*, *34*(11), pp.1807-1815.

Reviewer #2 (Remarks to the Author):

This is a strong and interesting manuscript, and the core idea of explicitly differentiating upper and lower NADW to obtain a more dynamical view of AMOC strength changes and their mechanisms is both timely and valuable. The focus on how u-NADW and l-NADW respond differently to perturbations, and how this links to Gulf Stream migration, significantly advances process-level understanding beyond bulk AMOC metrics.

Overall view:
The manuscript presents high-resolution sediment records from the Northwest Atlantic margin to reconstruct coordinated changes in u-NADW, l-NADW, and Gulf Stream position during the last deglaciation, with an emphasis on the Younger Dryas. The explicit separation of u-NADW and l-NADW responses, and the proposed sequence of circulation changes leading atmospheric and cryospheric responses, is conceptually powerful and aligns well with modern observational work that treats upper and lower NADW as dynamically distinct layers. The writing is generally clear, the multiproxy strategy is compelling, and the topic is highly relevant for understanding both past abrupt climate events and future AMOC behavior.

We thank the reviewer for their supportive comments.

My concerns and related question are listed below:
1. From local sortable silt to u-NADW strength: the logical step from “local near-bottom flow speed at ~1–2 km” to “basin-scale u-NADW strength” remains somewhat under-argued and could be made more robust.
a. The text currently assumes that, because the cores lie today within the u-NADW layer and beneath the Deep Western Boundary Current, changes in sortable silt necessarily reflect changes in u-NADW strength. It would help to more explicitly consider and, where possible, rule out alternative contributors to local flow variability, such as mesoscale recirculation, slope current variability, or changes in overlying Gulf Stream path and eddy activity that could alter shear and near-bottom stress without requiring a basin-wide change in u-NADW formation. Bruera et al. (2023) might provide some insight.
b. The authors could better explain why the chosen sites are expected to be particularly sensitive to changes in the strength of the u-NADW limb rather than more local circulation changes, perhaps by drawing on modern hydrographic/velocity sections or model output that show the dominance of the u-NADW layer at these depths and locations.

We thank the reviewer for their constructive suggestions (note that, given the overlap between comments a and b, we provide a joint response to these points). We agree that it is helpful to more explicitly rule out alternative contributors to grain size variability and thank the reviewer for the constructive suggestion to include modern hydrographic and velocity sections to support our interpretation. We have therefore incorporated modern hydrographic data from the EN4.2.1 dataset, as well as velocity sections first published by Pickart & Smethie (1998) and Bower & Hunt (2000).

- We rule out the influence of deeply penetrating slope waters, as modern hydrographic sections show that our cores are located between isopycnals corresponding to the upper and lower bounds of upper North Atlantic Deep Water (u-NADW).
- Conversely, velocity sections at 55°W and 60°W show that, at depths corresponding to our core sites, both locations lie within the core of fast flow associated with modern u-NADW.
- We rule out the influence of mesoscale recirculation as an increase in the northern recirculation gyre would cause a southward shift in Gulf Stream, inconsistent with our inferred northward shift in Gulf Stream.
- We also explicitly discuss the potential influence of eddy activity. Although a recent model study (van Westen & Dijkstra, 2026) suggests that a northward shift in Gulf Stream could lead to an increase in eddy kinetic energy in Northwest Atlantic shelf region, such a mechanism would require a near-total collapse of the Labrador Current and u-NADW. We consider this mechanism implausible for two main reasons: (1) A wide range of additional regional evidence from both upstream and downstream areas suggests a strengthening u-NADW formation and ventilation; and (2) the distinct phase lag between our upper ocean warming and increase in $\overline{SS}$ provides further support because an enhanced eddy kinetic energy would require a collapse in Labrador Current, which would instead produce synchronous increase in eddy kinetic energy and upper ocean warming.

We present these hydrographic and velocity sections in Supplementary Fig. 1 (below) and Fig. 1 (see above the responses to Reviewer 1), respectively, and have also added relevant text to the section *"Seesaw relationship between the upper- and lower-North Atlantic Deep Water"*, as detailed below:

"An important consideration is whether our SS records reflect changes in basin-scale u-NADW strength rather than other oceanographic processes, such as slope-water variability, mesoscale recirculation, or eddy activity. Modern hydrographic data from the Northwest Atlantic (Supplementary Fig. 1) show that both 09GGC and 44GGC lie within the density layer occupied by u-NADW, rather than being influenced by deeply penetrating slope waters. Consistent with this, modern velocity sections (Fig. 1c, d) indicate that our sites lie within the core of the southward-flowing u-NADW within the Deep Western Boundary Current. An alternative explanation for the observed increase in $\overline{SS}$ during the YD could be a strengthening of the northern recirculation gyre. However, model results suggest that a stronger recirculation gyre would shift the Gulf Stream southward[42], opposite to the behaviour reconstructed from our records (see section below).

Another potential driver for the observed increase in our $\overline{SS}$ records is an enhanced eddy kinetic energy[37] given the proximity of strong frontal systems and associated baroclinic instability (Fig. 1a). As the Labrador Current and u-NADW act as a barrier for ocean eddies near the slope, an enhanced eddy activity would require a near-collapse of the Labrador Current and u-NADW[43]. However, this mechanism is inconsistent with a wide range of additional regional evidence that supports the prevailing paradigm[14] of more vigorous u-NADW formation and ventilation during the YD: (1) Published $\overline{SS}$ data also reveal faster flow of u-NADW at ~1 km water depth at Flemish Pass[44] and at 1.5-2 km water depth on the slope to the west and north of Flemish Cap[45,46], as well as more vigorous flow of u-NADW (sometimes also termed glacial North Atlantic Intermediate Water) in the subtropical Northwest Atlantic[47] and subpolar Northeast Atlantic[48]; (2) benthic-planktic foraminiferal radiocarbon data indicate better ventilated intermediate water at 1.5 km water depth at the Tail of the Grand Banks (Fig. 3c)[34], and well-ventilated water above 2.3 km in the subtropical Northwest Atlantic[49]; (3) Cd/Ca data from the Bahama Banks indicate the enhanced production of nutrient-depleted North Atlantic intermediate water during the YD[18]. Taken together, these observations support the interpretation that our SS records reflect basin-scale variability in u-NADW strength rather than local circulation changes."

We have also added the following text in the section *"Phasing of ocean reorganization during the Younger Dryas onset and its implications"* to provide further support for the interpretation that our sortable silt data do not reflect changes in eddy kinetic energy:

"Critically, the distinct phase lag between our %Np and $\overline{SS}$ records provides further support for the interpretation that the observed increase in $\overline{SS}$ is not driven by changes in eddy activity. Such a mechanism would require a near-collapse of the Labrador Current[43], which would instead lead to synchronous upper ocean warming (i.e. decrease in %Np) and enhanced eddy kinetic energy (i.e. increase in $\overline{SS}$)."

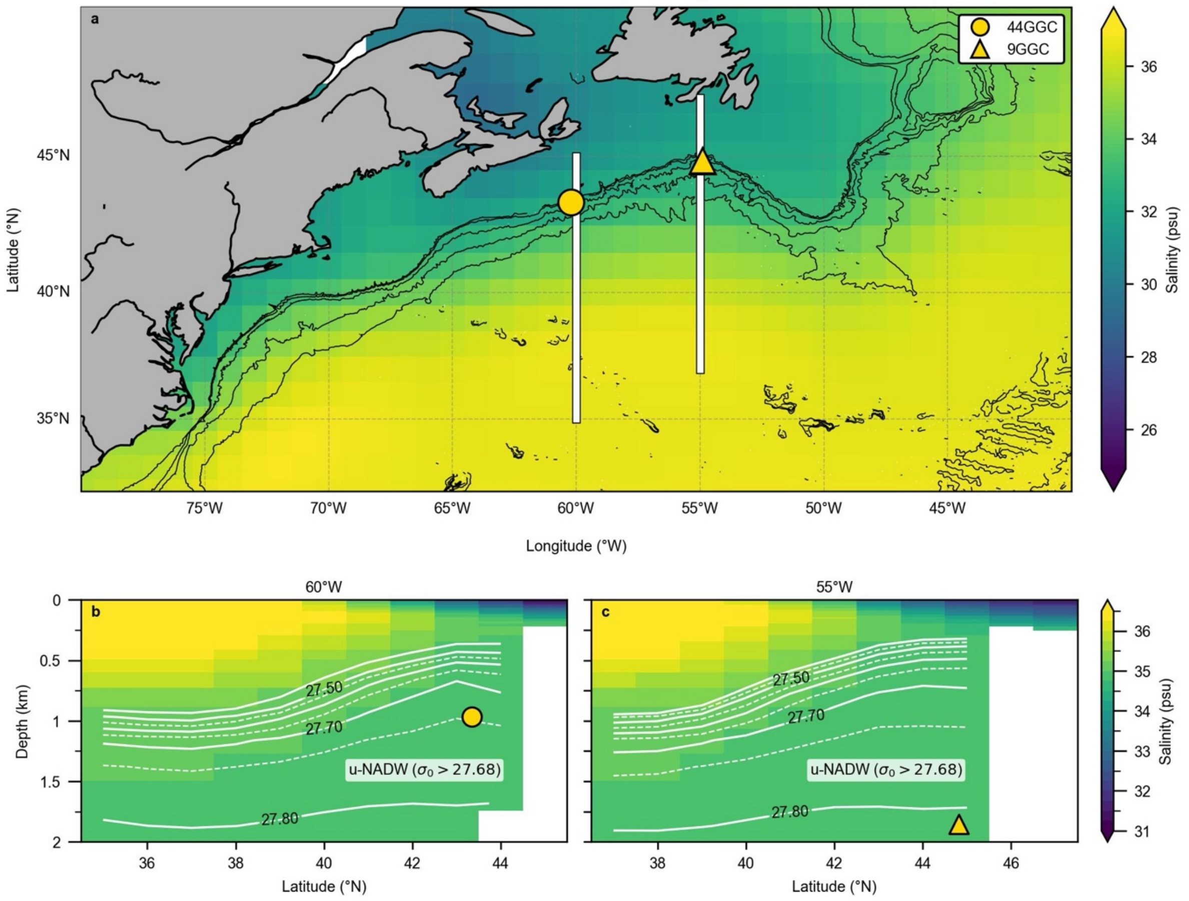

Supplementary Fig 1. *Salinity (colour shading) and potential density (σ_0, kg m^{-3}; white contours) in the northwest Atlantic derived from EN4.2.1, averaged between January 2000 and January 2020.* ***a*** *Sea-surface salinity with bathymetric contours at 0.5, 1, 2, 3, and 4 km water depth (black contours).* ***b-c*** *Salinity and potential density sections at 60°W (**b**) and 55°W (**c**). Solid white lines in **a** indicate the locations of the zonal sections shown in **b** and **c**. Yellow symbols mark the locations of the sediment core sites, both of which are situated at depths currently bathed by waters with densities characteristic of upper North Atlantic Deep Water (u-NADW; σ_0 > 27.78 kg m^{-3})[8] (Note that density value associated with 09GGC is marginally high due to the coarse resolution of the EN4 dataset.).This indicates that the sites are influenced by u-NADW rather than deeply penetrating slope waters, implying that the sortable-silt mean grain-size reconstructions are representative of u-NADW flow speeds. EN4 provides monthly, gridded objective analyses of available ocean observations at 1° horizontal resolution[9]; fields and sections were extracted by time-averaging salinity and temperature for the 2000-2020 period. Density was computed using the Python implementation of the Gibbs SeaWater Oceanographic Toolbox[10].*

c. Given that the seesaw between u- and l-NADW is a central result, a short sensitivity discussion (or schematic) clarifying how much of the observed sortable silt signal would be expected from plausible changes in local circulation versus true u-NADW transport changes would strengthen the causal inference.

We agree with the reviewer that it would strengthen the causal inference to quantify the likely contribution of u-NADW transport versus local circulation to our flow speed records.

However, quantifying u-NADW transport would require a large array of carefully selected transects (e.g., locations unaffected by recirculation), which is challenging even for modern physical oceanographic data. Therefore, while we agree that quantifying u-NADW transport would be highly valuable, this lies beyond the scope of the present study but represents an important avenue for future work. Instead, we draw on a range of different proxies that qualitatively indicate an overall strengthening of the u-NADW during the YD, including grain size data from our study and a number of proxy records (e.g., grain size; benthic-planktic radiocarbon data; Cd/Ca data) from published studies. Future work is required to quantitatively reconstruct the u-NADW transport by increasing the spatial coverage of data in this crucial transition region.

Furthermore, our study indicating a strengthened u-NADW during the YD also sets a motivated target for future quantitative analysis using emerging high-resolution paleoclimate models since current generation of transient simulations for the last deglaciation (e.g., TraCE-21ka) do not have sufficient resolution to fully resolve the Deep Western Boundary Current, thereby precluding quantification of u-NADW transport at present.

2. Interpreting local margin warming as Gulf Stream latitude shifts: the current argument of a northward shift of the Gulf Stream is plausible and supported by the similarity to modelled and observed SST "dipole" patterns under AMOC weakening. Nevertheless, the chain of reasoning from local thermal anomalies to meridional Gulf Stream migration could be more critically examined.
a. The manuscript leans heavily on spatial analogies to modern SST dipoles and model experiments, plus the present-day proximity of the sites to the Gulf Stream front. It would be valuable to discuss more explicitly whether changes in eddy heat fluxes, shelf-slope current structure, or along-slope advection could, in principle, produce similar local warming at these cores without a large-scale meridional shift of the Gulf Stream path. This once again is a rule out exercise.
The reason being, in Molodtsov et al. (2025), observed multidecadal changes in North Atlantic temperature and salinity can be largely explained by external forcings (greenhouse gases, aerosols, volcanic eruptions, etc.), and that CMIP6 models tend to underestimate the amplitude or structure of these forced signals. This means that what might look like internally driven AMOC variability or purely "ocean-led" changes here can, in fact, be strongly shaped by the time-varying external forcing, making causal inferences based solely on internal dynamics potentially misleading.
b. If available, additional independent indicators of frontal position (e.g., faunal assemblages that track front location, or comparison with cores straddling the modern Gulf Stream path) could be highlighted more strongly, or the limits of reconstructing exact Gulf Stream latitude from the present data could be acknowledged.

We thank the reviewer for their thoughtful suggestions (given the overlap between comment a and b, we again provide a joint response to these comments). We agree that it is helpful to more explicitly rule out alternative contributors to local warming. We consider a weakening Labrador Current as a potential driver for the warming recorded in our sites by reducing the advection of cold water and enhancing eddy activity. As discussed above, we do not favour this interpretation given a wide range of regional

evidence suggesting a strengthening Labrador Current and u-NADW during the YD. We also agree it would be useful to reconstruct the exact Gulf Stream latitude using additional independent indicators. However, due to the scarcity of cores straddling the modern Gulf Stream path, we cannot precisely constrain the frontal location at present. Instead, we added an additional supplementary figure (below) to compare our temperature records with site further south at Bermuda Rise (Carlson et al., 2008), where we show that the warming recorded in our cores are not accompanied by warming in the subtropical latitudes, suggesting that it is not driven by a basin-wide warming. Together with the SST dipole we show in Fig. 4, this supports the interpretation that the warming in the Northwest Atlantic is driven by Gulf Stream frontal shifts. We have therefore expanded our discussion in the Section *"Surface-subsurface ocean warming: Northward Gulf Stream shift"* as detailed below:

"Several other mechanisms could also influence upper ocean temperature in the Northwest Atlantic shelf region, such as increased eddy heat flux, influence of along-slope advection, and basin-wide changes likely associated with external forcing. A weakening Labrador Current could in principle drive warming at our core sites by reducing the advection of relatively cold water and enhancing local eddy activities[43]. However, a wide range of evidence instead shows a strengthened Labrador Current and u-NADW during the YD[18,34,44–49] (Fig. 3a-c). Furthermore, the absence of ocean warming in the subtropical Northwest Atlantic[76] precludes a basin-wide warming during the YD (Supplementary Fig. 10). Therefore, we favour the interpretation that the surface-subsurface warming in the Northwest Atlantic shelf region is driven by northward migration of the Gulf Stream. Future work is required to precisely constrain the Gulf Stream location by increasing the spatial coverage of core materials from this region."

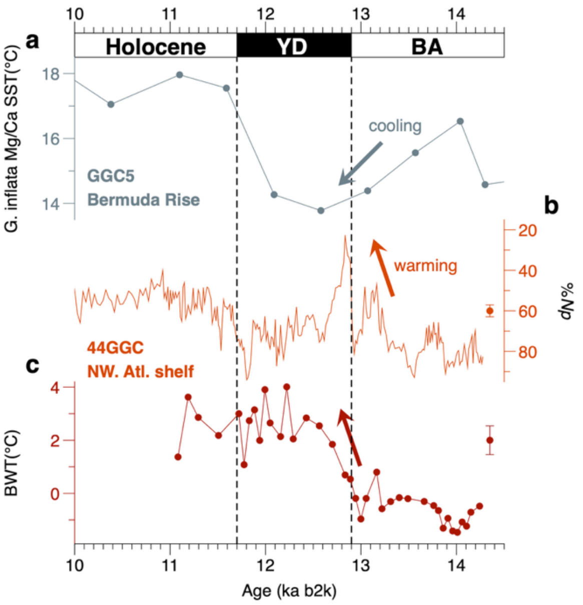

Supplementary Fig 10. Comparison of ocean temperature records between Northwest Atlantic shelf and subtropical Northwest Atlantic. ***a*** *G. inflata Mg/Ca derived SST[22] of GGC5 from Bermuda Rise in the subtropical Northwest Atlantic.* ***b*** *%Np of 44GGC (this study).* ***c*** *44GGC benthic Mg/Ca derived bottom water temperature (BWT). In* ***b*** *and* ***c****, error bars show averaged ±1σ error.*

c. It would help to explicitly discuss how robust the inferred 480-year and ~800–900-year lags are to plausible end-member reservoir-age and tie-point choices. For instance, what range of lag remains if one considers conservative bounds on reservoir age variability or alternative IRD–Ca^{2+} alignments?

We agree that it is beneficial to explicitly discuss the robustness of the inferred phase lag at the onset of the YD. Because the inferred phase relationship is derived from the same core (see Supplementary Fig. 11 below), the uncertainty in the inferred lags primarily depends on the duration of the transition interval at the YD onset, which in turn reflects uncertainty in the sedimentation rate of the core. We therefore revised the mid-point analysis to incorporate age model uncertainties in the analysis:

"Mid-point analysis at the Younger Dryas onset

To determine the timing of the YD onset for each proxy record from core 44GGC (Fig. 5a-c), we defined the transition mid-point as the time at which the proxy reached the half-amplitude level, calculated as the midpoint between the maximum and minimum values within the interval 13.00-12.65 ka b2k. Because all proxy records are derived from the same sediment core and share a common age model (Supplementary Fig. 11), absolute age uncertainties are largely cancelled out when assessing relative phasing. We therefore propagated relative chronological uncertainty between tie points by applying 10,000 bootstrap realizations of stochastic linear stretching and compression of the transition window (13.00-12.65 ka b2k), constrained by ±1σ age uncertainties at the bounding ages. For each realization, the proxy records were linearly interpolated onto a uniform 1-year time step and the mid-point age was recalculated. Lead-lag distributions were then derived from the ensemble of warped time axes."

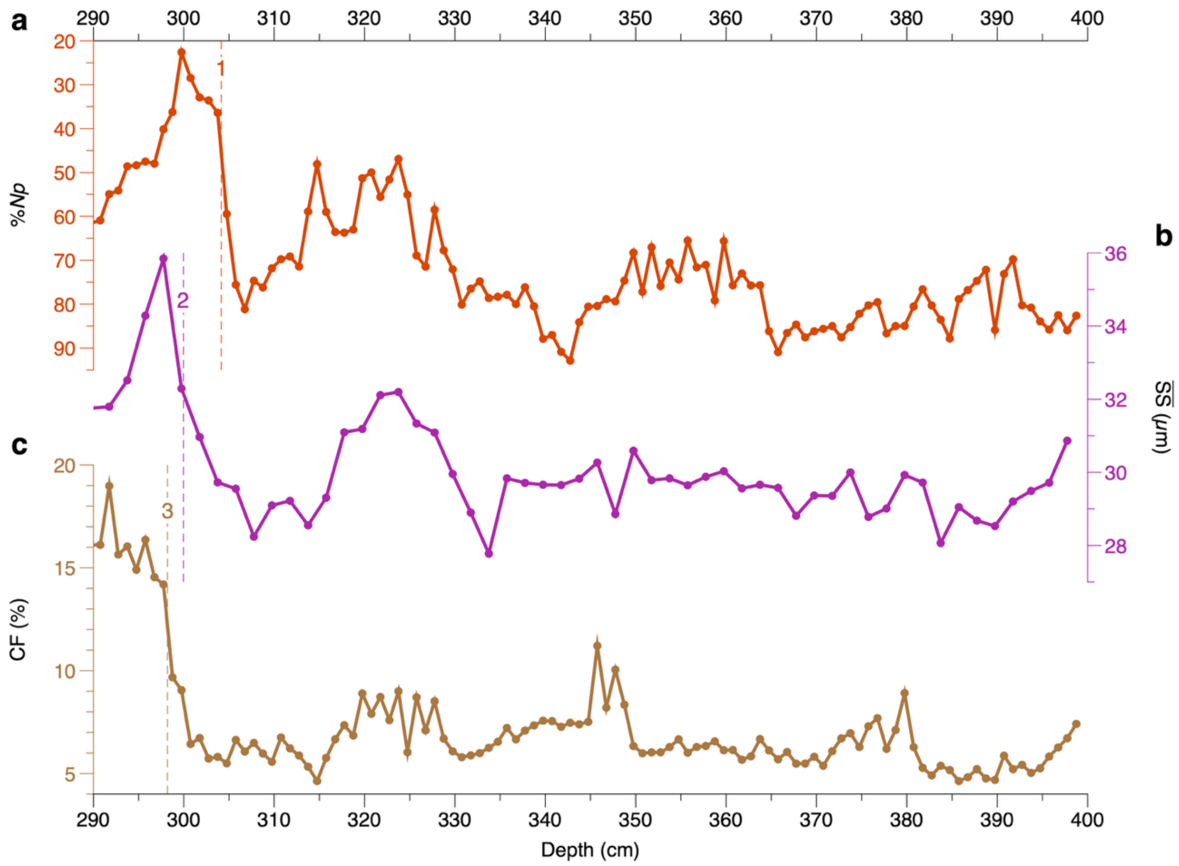

Supplementary Fig 11. Mid-point analysis at the YD onset (data plotted on depth). ***a*** *%Np.* ***b*** $\overline{SS}$ *measured on Coulter.* ***c*** *Percentage of coarse fraction (a proxy for IRD; Methods). Vertical dashed lines in* ***a-c*** *indicate the mean depth of the mid-point for BA-YD transition for each proxy derived from bootstrapping analysis (Methods).*

References:
Molodtsov, S., Marinov, I., Weijer, W. et al. North Atlantic temperature and salinity changes are driven by external forcing, underestimated by CMIP6 models. npj Clim Atmos Sci 8, 332 (2025). https://doi.org/10.1038/s41612-025-01210-w
Bruera, R., Curbelo, J., García-Sánchez, G., & Mancho, A. M. (2023). Mixing and geometry in the North Atlantic Meridional Overturning Circulation. Geophysical Research Letters, 50, e2022GL102244. https://doi.org/10.1029/2022GL102244

References
van Westen, R.M. and Dijkstra, H.A., 2026. Abrupt Gulf Stream path changes are a precursor to a collapse of the Atlantic Meridional Overturning Circulation. *Communications Earth & Environment*, *7*(1), p.197.

Response to referees: “Co-ordinated Shifts in Deep-water Formation and Gulf Stream Migration During Abrupt Climate Changes” by Fangjingcheng Zhu, Alice Carter-Champion, Jack H. Wharton, Joel Bracamontes-Ramírez, Andrea Burke, Peter B. de Menocal, David Fairman, Lloyd D. Keigwin, Thomas M. Marchitto, Eirini Papachristopoulou, James W. B. Rae, Yair Rosenthal, Ning Zhao, and David J. R. Thornalley.

Author’s note – our detailed responses are given below in blue, with relevant direct quotes from the manuscript included where appropriate (*blue italics*). We have also included updated figures where appropriate.

Reviewer #1 (Remarks to the Author):

The authors have carefully addressed all my concerns from the first round of reviews. These changes have greatly improved the flow and clarity of the manuscript. I only have a few of typographical suggestions:

1. Line 89: “shown in black dots” should be “shown as black dots” Corrected.
2. Fig. 5e: the “Sea ice expansion” arrow is slightly overlapping Corrected.
3. Throughout the manuscript ranges should use en dashes instead of hyphens (e.g., Line 680 and 784)

We thank the reviewer for this helpful suggestion and have carefully reviewed the entire manuscript, replacing hyphens with en dashes in all ranges.

Thank you for taking the time to thoroughly address all my comments.

We are very grateful for the reviewer’s constructive suggestions, which have greatly improved our manuscript.

Reviewer #2 (Remarks to the Author):

The authors have substantially improved the manuscript and, in my view, have satisfactorily addressed the major issues raised in the first round, both by myself and by the other reviewer. The revised paper is now much clearer in its treatment of chronology, the linkage between sortable silt and u NADW strength, and the interpretation of Gulf Stream migration and phasing.

We thank the reviewer for their supportive comments.

In my view, the revised explanation for why the sortable silt records reflect changes in u NADW strength is now broadly convincing and adequate for this journal, but it still rests on several reasonable assumptions that should remain explicit. The authors have done a good job showing, with modern hydrography and velocity sections, that the cores sit in the present day u NADW density/velocity core rather than in slope water, and they carefully argue against alternative local controls such as slope water variability, recirculation changes, or enhanced eddy activity, supported by the coherence with

independent regional proxies. At the same time, interpreting these boundary current records as indicative of basin scale u NADW and AMOC changes necessarily involves extrapolating from a limited set of sites and assuming that late deglacial water mass structure is sufficiently analogous to the modern. I am comfortable with these inferences, but I would encourage the authors to keep the wording slightly cautious (e.g. "we infer", "our records are consistent with basin scale changes") when referring to u NADW and AMOC at the basin scale, to signal to readers that the conclusions are built from strong regional evidence plus physically motivated, but not directly observed, assumptions.

We thank the reviewer for the constructive suggestions and agree that it is helpful to keep the wording slightly more cautious when referring to u-NADW/AMOC at basin scale given that our conclusions are built from strong regional evidence and physically motivated assumptions rather than direct observations. We have therefore carefully reviewed the manuscript and kept the wording more cautious (highlighted in yellow shadings in the revised manuscript).

My remaining comments are relatively minor and mostly relate to wording and scope:

• Similarly, the discussion of a "northward shift" of the Gulf Stream is now much better justified, but the data most robustly demonstrate an increased influence of Gulf Stream–derived waters and a dipole-like pattern, rather than a uniquely determined meridional path change. It may be helpful to emphasize that, with current core coverage, the results point to a northward displacement and/or strengthening of the Gulf Stream influence in this region, and that finer details of path geometry must await denser spatial sampling.

We agree with the reviewer that it is helpful to emphasize the limitation of current core coverage and the need for denser spatial sampling in future work. We have therefore added the following sentence in our discussion:

"While our results point to a northward displacement and/or strengthening of the Gulf Stream influence in this region, future work is required to precisely constrain the finer details of Gulf Stream path geometry by increasing the spatial coverage of core materials from this region."

• The extension of the deglacial mechanism to industrial era and future AMOC changes is interesting and well signposted as a hypothesis. I would just recommend ensuring that this forward-looking discussion is clearly distinguished from the core, data driven deglacial results (e.g. a brief reminder that the industrial-era interpretation remains to be tested with robust l NADW reconstructions).

We thank the reviewer for this thoughtful suggestion, and we agree that it is helpful to ensure a clear distinction between the forward-looking discussion and our data-driven deglacial results. We have therefore revised the following sentence:

"Testing the hypothesis that these co-ordinated industrial era shifts in North Atlantic surface ocean circulation can be attributed to a concomitant weakening of l-NADW – in a manner analogous to the deglacial events we have described here – will require the

development of robust historical records of l-NADW strength, as this interpretation remains to be directly tested, for which efforts are already underway."

Recommendation
Overall, I think the authors have done an excellent job of addressing the previous round of comments. The chronology is now treated with appropriate methodological rigor and transparency, the physical interpretation of the sortable silt and temperature proxies is much more tightly argued, and the causal narrative is expressed with a level of caution that matches the strength of the evidence. I am happy with the revisions and support publication after the authors make any minor wording adjustments they and the editor deem necessary.

We thank again for the supportive comments, and we greatly appreciate all the constructive suggestions from the reviewer that have greatly improved our manuscript.

In addition, we have moved our original Supplementary Fig. 7 (Surface ocean stratification regime reconstructed using $\Delta\delta^{18}O_{Nps\text{-}Gb}$ of 44GGC) to Fig. 6 in the main manuscript to improve the clarity of the paper, in line with the journal's requirement with up to 10 display items in the main article.
